# Cytokinins regulate spatially specific ethylene production to control root growth in *Arabidopsis*

**DOI:** 10.1016/j.xplc.2024.101013

**Published:** 2024-07-03

**Authors:** Amel Yamoune, Marketa Zdarska, Thomas Depaepe, Anna Rudolfova, Jan Skalak, Kenneth Wayne Berendzen, Virtudes Mira-Rodado, Michael Fitz, Blanka Pekarova, Katrina Leslie Nicolas Mala, Paul Tarr, Eliska Spackova, Lucia Tomovicova, Barbora Parizkova, Abigail Franczyk, Ingrid Kovacova, Vladislav Dolgikh, Elena Zemlyanskaya, Marketa Pernisova, Ondrej Novak, Elliot Meyerowitz, Klaus Harter, Dominique Van Der Straeten, Jan Hejatko

**Affiliations:** 1 CEITEC (Central European Institute of Technology), Masaryk University, Brno, Czech Republic; 2 National Centre for Biomolecular Research, Faculty of Science, Masaryk University, Brno, Czech Republic; 3 Laboratory of Functional Plant Biology, Department of Biology, Ghent University, Gent, Belgium; 4 Center for Plant Molecular Biology, University of Tübingen, Tübingen, Germany; 5 Howard Hughes Medical Institute and Division of Biology and Biological Engineering, California Institute of Technology, Pasadena, CA, USA; 6 Faculty of Science, Palacký University and Institute of Experimental Botany, The Czech Academy of Sciences, Olomouc, Czech Republic; 7 Institute of Cytology and Genetics, Siberian Branch, Russian Academy of Sciences, Novosibirsk 630090, Russia; 8 Faculty of Natural Sciences, Novosibirsk State University, Novosibirsk 630090, Russia

**Keywords:** cytokinin, ethylene, ACC SYNTHASE, ACC OXIDASE, multistep phosphorelay, *Arabidopsis*

## Abstract

Two principal growth regulators, cytokinins and ethylene, are known to interact in the regulation of plant growth. However, information about the underlying molecular mechanism and positional specificity of cytokinin/ethylene crosstalk in the control of root growth is scarce. We have identified the spatial specificity of cytokinin-regulated root elongation and root apical meristem (RAM) size, both of which we demonstrate to be dependent on ethylene biosynthesis. Upregulation of the cytokinin biosynthetic gene *ISOPENTENYLTRANSFERASE* (*IPT*) in proximal and peripheral tissues leads to both root and RAM shortening. By contrast, *IPT* activation in distal and inner tissues reduces RAM size while leaving the root length comparable to that of mock-treated controls. We show that cytokinins regulate two steps specific to ethylene biosynthesis: production of the ethylene precursor 1-aminocyclopropane-1-carboxylate (ACC) by ACC SYNTHASEs (ACSs) and its conversion to ethylene by ACC OXIDASEs (ACOs). We describe cytokinin- and ethylene-specific regulation controlling the activity of *ACSs* and *ACOs* that are spatially discrete along both proximo/distal and radial root axes. Using direct ethylene measurements, we identify *ACO2*, *ACO3*, and *ACO4* as being responsible for ethylene biosynthesis and ethylene-regulated root and RAM shortening in cytokinin-treated *Arabidopsis*. Direct interaction between ARABIDOPSIS RESPONSE REGULATOR 2 (ARR2), a member of the multistep phosphorelay cascade, and the C-terminal portion of ETHYLENE INSENSITIVE 2 (EIN2-C), a key regulator of canonical ethylene signaling, is involved in the cytokinin-induced, ethylene-mediated control of *ACO4*. We propose tight cooperation between cytokinin and ethylene signaling in the spatially specific regulation of ethylene biosynthesis as a key aspect of the hormonal control of root growth.

## Teaser

This study provides evidence demonstrating that cytokinin-regulated root growth control is dependent on the spatially specific activation of ethylene biosynthesis. Cytokinins upregulate both ACC SYNTHASEs and ACC OXIDASEs in a cytokinin- and/or ethylene-specific manner. Cytokinin-responsive multistep phosphorelay and canonical ethylene signaling cooperate to upregulate ethylene biosynthesis.

## Introduction

Roots or root-like structures are one of the key adaptations of plants that were critical for terrestrial colonization (Hetherington and Dolan, 2018). Roots mediate a number of biotic and abiotic interactions (Comas et al., 2013; Bakker et al., 2018), and root architecture is one of the key yield-determining traits under both normal and stress (particularly drought) conditions (Lynch, 2007; Uga et al., 2013; Ramireddy et al., 2018). Understanding the factors that control root growth is critical for building a comprehensive picture of plant developmental and adaptive responses that directly impact crop productivity.

The overall growth rate of the root is determined by the balance between three fundamental processes: i) cell proliferative activity in the root apical meristem (RAM), ii) cell differentiation, and iii) elongation of cells leaving the RAM. All of these processes are known to be under the control of phytohormones, including cytokinins and ethylene (reviewed by Takatsuka and Umeda, 2014; Kong et al., 2018; Svolacchia et al., 2020; Yamoune et al., 2021). Cytokinins control the size and proliferation capacity of the RAM both in a positive and negative way. Cytokinins increase RAM size by enhancing stem cell proliferation but can also shorten the RAM (a process involving crosstalk with auxin and gibberellic acid) by inducing cell differentiation in the root transition zone (for a recent review see Svolacchia et al., 2020; Yamoune et al., 2021). The involvement of cytokinin-regulated auxin transport has been invoked in the regulation of root cell elongation in both an ethylene-dependent and -independent manner (Street et al., 2016), possibly by induction of cell wall stiffening (Liu et al., 2022).

Ethylene is one of the main regulators of root cell elongation, with an inhibitory effect that has been known for decades (Dolan, 1997). This ethylene-mediated inhibition of cell elongation is not limited to the root and is to a large extent, if not exclusively, dependent on ethylene-regulated auxin biosynthesis and transport (Stepanova and Alonso, 2009; Hu et al., 2017; Vaseva et al., 2018; Zemlyanskaya et al., 2018; Mazzoni-Putman et al., 2021). Continuous treatment with the ethylene biosynthesis precursor 1-aminocyclopropane-1-carboxylate (ACC) leads to the inhibition of cell elongation by repressing cell elongation-promoting factors and inducing genes whose products attenuate cell elongation (Markakis et al., 2012). Despite this, the early ethylene response can be both positive and negative (depending on the developmental context and its position in the RAM epidermis), and this effect seems to be independent of the role of ethylene in inducing cell differentiation (Le et al., 2001). Apart from its role in cell elongation, ethylene has also been shown to control cell division in the root stem cell niche (Ortega-Martinez et al., 2007) and participates (along with cytokinins) in the control of RAM size by inducing cell differentiation in the root transition zone (Street et al., 2015; Zdarska et al., 2019).

Ethylene biosynthesis in plants starts with the conversion of methionine by S-adenosyl-L methionine synthetase into S-adenosyl methionine, the general ethylene precursor shared by several metabolic pathways. SAM serves as a substrate for ACC SYNTHASEs (ACSs), mediating the first (and rate-limiting) step dedicated exclusively to ethylene biosynthesis, leading to formation of the non-proteinogenic three-membered-ring amino acid 1-aminocyclopropane-1-carboxylic acid (ACC). ACC oxidation to ethylene by ACC OXIDASEs (ACOs) is the second and final step specific to the ethylene biosynthetic pathway. Given the key importance of ethylene in many aspects of the plant life cycle, it is not surprising that the activities of both ACSs and ACOs are under tight transcriptional and posttranscriptional control. Moreover, levels of the non-proteinogenic amino acid ACC can be further regulated by conjugation and translocation. For more detailed information on ethylene biosynthesis, see recent reviews by Depaepe and Van Der Straeten (2020) and Pattyn et al. (2021).

Ethylene is perceived by the ethylene-responsive sensor histidine kinases ETHYLENE RESPONSE 1 (ETR1) and ETHYLENE RESPONSE SENSOR 1 (ERS1) and by the HK-like Ser/Thr kinases ETR2, ERS2, and ETHYLENE INSENSITIVE 4 (EIN4) (reviewed in Chen et al., 2005; Etheridge et al., 2006; Binder, 2020). The downstream target of ER-located ethylene sensors in the canonical ethylene signaling pathway is the Raf family Ser/Thr kinase CONSTITUTIVE TRIPLE RESPONSE 1 (CTR1) (Kieber et al., 1993). Both the receptors and CTR1 act as negative regulators of the signaling pathway. Ethylene binding switches off the ethylene sensors, attenuating CTR1-mediated phosphorylation of the ER-associated N-ramp-like protein EIN2. As a result, the C-terminal end of hypo-phosphorylated EIN2 (EIN2-C) is disinhibited and cleaved off. In the cytoplasm, EIN2-C initiates degradation of the mRNA of *EIN3-BINDING F BOX PROTEIN* (*EBF1*) and *EBF2*, leading to stabilization of the ethylene-responsive transcription factor EIN3. In parallel, EIN2-C translocates into the nucleus, becoming part of the complex that facilitates EIN3-regulated transcription (Ju and Chang, 2012; Wen et al., 2012; Li et al., 2015; Binder, 2020).

Cytokinin signaling is also initiated by histidine kinases, but the downstream response, unlike that for ethylene, is mediated via a multistep phosphorelay (MSP) pathway, also called two-component signaling (for a review see Kieber and Schaller, 2018; Mira-Rodado, 2019; Leuendorf and Schmuelling, 2021). In the MSP pathway, cytokinins are perceived by the CHASE domain of ARABIDOPSIS HISTIDINE KINASE 2 (AHK2), AHK3, and AHK4, leading to autophosphorylation of a conserved His. This triggers the His-to-Asp-to-His-to-Asp downstream phosphorelay and activation (via phosphorylation of their conserved Asp residue) of nuclear-localized type B ARABIDOPSIS RESPONSE REGULATORs (RRBs; Heyl et al., 2013), which act as cytokinin-regulated transcription factors.

Regulation of root growth involves tight cytokinin/ethylene crosstalk. Exogenous cytokinins have an inhibitory effect on root cell elongation (Beemster and Baskin, 2000) that is mediated by cytokinin-induced ethylene production; the inhibitory effect of cytokinins on the elongation of both root and hypocotyl cells was shown to depend on functioning ethylene signal transduction (Cary et al., 1995; Ruzicka et al., 2009). In line with that finding, the regulatory effect of cytokinins on both RAM size and root cell elongation in rice was shown to be mediated by increased ethylene content (Zou et al., 2018). Mechanistically, cytokinin and ethylene interact at the level of both biosynthesis and signaling. Tight interaction between MSP and canonical ethylene signaling has been reported (for a recent review, see Skalak et al., 2021). In brief, ETR1 was shown to mediate ethylene-regulated MSP signaling in the root transition zone to control RAM size via ethylene-induced cell differentiation (Street et al., 2015; Zdarska et al., 2019). The action of ETR1 was proposed to be mediated via ETR1-induced phosphorylation of the histidine kinase AHK5 (Szmitkowska et al., 2021), eventually leading to phosphorylation of the RRB ARR2 (Hass et al., 2004). In rice, the ethylene sensor OsERS2 was shown to interact with the AHK5 ortholog MHZ1/OsHK1 and control its HK activity (Zhao et al., 2020). Cytokinins were also demonstrated to upregulate ethylene biosynthesis by stabilizing ACS5 and ACS9 (Vogel et al., 1998; Chae et al., 2003; Rashotte et al., 2005; Hansen et al., 2009).

Here, we describe the identification of a tight interaction network between cytokinins and ethylene biosynthetic genes. We show that cytokinins not only stimulate ACC production by transcriptional regulation of several ACSs but also regulate the last step of ethylene biosynthesis by activating ACOs. We show that cytokinins control root elongation and RAM size by inducing ethylene biosynthesis in a spatially distinct manner, and we describe a novel mechanism of interaction between MSP and canonical ethylene signaling that drives the expression of *ACO4*.

## Results

### Cell-type-specific cytokinin overproduction is necessary to induce ACC biosynthesis and root shortening

**Figure 1:**
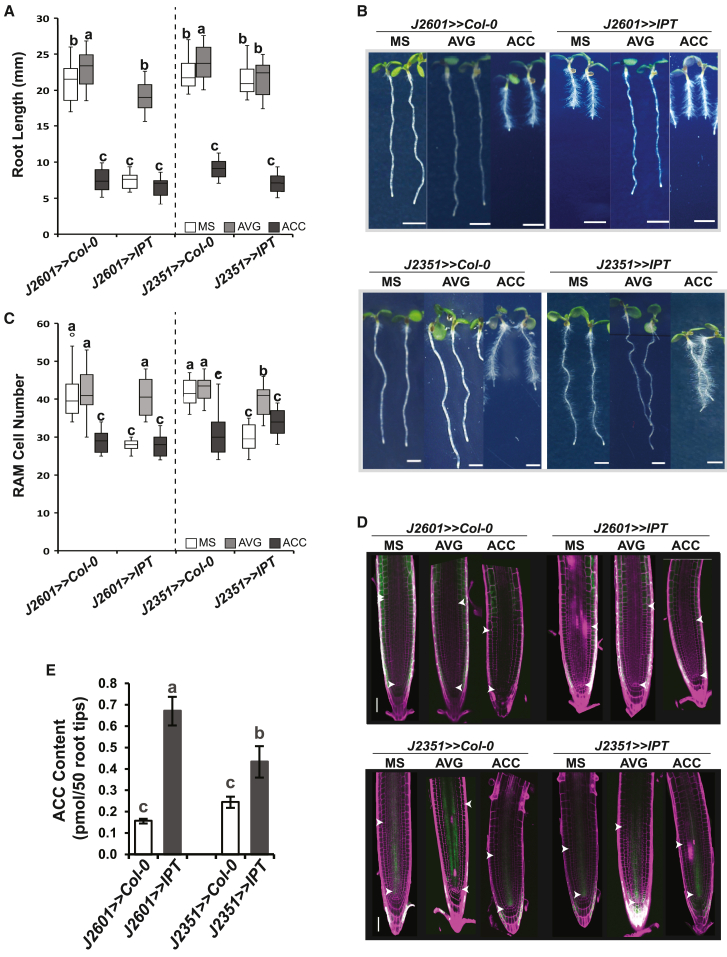
Ectopic overexpression of *IPT* in the epidermis induces root shortening. **(A–D)** Root length **(A)**, RAM size **(B),** and representative images **(C and D)** of 6-day-old seedlings of *IPT*-overexpressing lines: *J2601>>IPT* (epidermis/cortex) and *J2351>>IPT* (stele/LRC) and their respective controls *J2601>>Col-0* and *J2351>>Col-0* grown on ½ MS media supplemented or not with 0.2 μM AVG or 1 μM ACC. **(E)** ACC levels in root tips of *J2601>>IPT* and *J2351>>IPT* lines and their respective controls *J2601>>Col0* and *J2351>>Col0*. The boxplots in **(A)** and **(B)** represent data from three independent replicates (*n* = 15), and the bars in **(E)** represent the means ± SD of three biological replicates; the letters indicate significance classes determined by a linear mixed model ANOVA and Tukey’s post hoc HSD test; in **(A)** and **(C)**, significance classes were calculated separately for *J2601>>Col0* together with *J2601>>IPT* and *J2351>>Col0* together with *J2351>>IPT*. The white arrowheads in **(D)** mark the extent of the RAM. Scale bars represent 2.5 mm in **(B)** and 100 μm in **(D)**.

Exogenously applied cytokinins inhibit root cell elongation primarily through cytokinin-induced ethylene production, as cytokinin-induced root shortening depends on functional ethylene signaling or ethylene biosynthesis (Ruzicka et al., 2009 and Supplemental Figure 1). To assess the potential cell-type specificity of cytokinin-induced inhibition of root elongation, we upregulated cytokinin biosynthesis in the outer RAM cell layers (epidermis and cortex), shown to be required for ethylene-regulated root growth (Vaseva et al., 2018), and in the more internal (provascular/stele) tissues, suggested to be important for cytokinin-mediated RAM shortening (Dello Ioio et al., 2007). This was achieved by activating the cytokinin biosynthetic gene *ISOPENTENYLTRANSFERASE* (*IPT*) in a cell-type-specific manner using the GAL4>>UAS activator–reporter system (Laplaze et al., 2005; Bielach et al., 2012). *IPT* upregulation in the epidermis/cortex of the root transition/elongation zone and in more proximal differentiated tissues in J2601>>*IPT* was associated with a strong inhibition of root growth and a stimulation of root hair formation (Figure 1A and 1B), neither of which was observed in the presence of 2-aminoethoxyvinylglycine (AVG), an inhibitor of ACC biosynthesis (Yang and Hoffman, 1984). By contrast, *IPT* activation in provascular tissues in J2351>>*IPT* resulted in no root length change and a weaker induction of root hair formation compared with *IPT upregulation in the* epidermis/cortex, although the roots were still sensitive to exogenously added ACC (Figure 1A and 1B). The root reduction observed in J2601>>*IPT* was accompanied by a comparably strong reduction in cell elongation, measured as the Length of the first fully differentiated Epidermal cell that showed a visible root Hair bulge (LEH; Le et al., 2001). This effect was not observable in the case of non-shortened roots of J2351>>*IPT*. By contrast, significant RAM reduction was observed upon *IPT* activation in both J2601>>*IPT* and J2351>>*IPT* (Figure 1C and 1D). Upregulation of *IPT* expression in the epidermis/cortex and in the provascular/stele tissues resulted in increased endogenous ACC levels. However, cytokinin-induced ACC upregulation was more pronounced in J2601>>*IPT* (4.3-fold change) than in J2351>>*IPT* (1.6-fold change; Figure 1E).

To support our findings on the spatial specificity of cytokinin-induced ethylene production and its functional importance for the inhibition of root elongation, we assayed the effects of exogenous cytokinin application on root growth of lines in which ethylene signaling was inhibited in specific cell types (Vaseva et al., 2018). We observed that lines overexpressing *EIN3-BINDING F BOX PROTEIN 2 (EBF2*), a negative regulator of ethylene signaling, in the outer cell files of the root, including the epidermis and lateral root cap (LRC; *pA14::EBF2* and *pLRC1::EBF2*) or multiple cell types including the epidermis (*p35S::EBF2*), were less sensitive to cytokinin-induced root shortening than the wild type (WT), Col-0. By contrast, lines in which ethylene signaling was attenuated in more internal and/or distal cell types, like the stele (*pS2::EBF2*), the proliferation zone of the RAM (*pCRH1::EBF2*), or the cortex of the root elongation zone (*pCOR::EBF2*), showed cytokinin sensitivity comparable to that of WT controls (Supplemental Figure 2A). All tested lines showed a statistically significant reduction in the cytokinin-induced reduction of cell elongation and RAM size (Supplemental Figure 2B and 2C). However, the strongest reduction in sensitivity to cytokinin-mediated inhibition of cell elongation was observed in lines that showed significantly reduced sensitivity to cytokinin-mediated root elongation (*pA14::EBF2*, *pLRC1::EBF2*, and *p35S::EBF2*; Supplemental Figure 2B).

In summary, our results confirm the tight interaction between cytokinins and ethylene biosynthesis in the control of root growth and show that cell-type-specific induction of cytokinin biosynthesis is important for cytokinin-induced ACC production and root shortening. Cytokinin upregulation in both distal/internal and proximal/outer tissues leads to RAM shortening, in which cytokinins and cytokinin-induced ethylene have an additive effect. However, the reduction in RAM size alone is not sufficient for significant inhibition of root elongation, as can be seen upon cytokinin upregulation in distal/internal tissues. By contrast, activation of endogenous cytokinin production in the proximal/outer cell types is necessary for cytokinin-induced, ethylene-mediated root growth reduction, mostly via inhibition of cell elongation.

### Both cytokinin and ethylene upregulate transcription of *ACC SYNTHASES*

**Figure 2:**
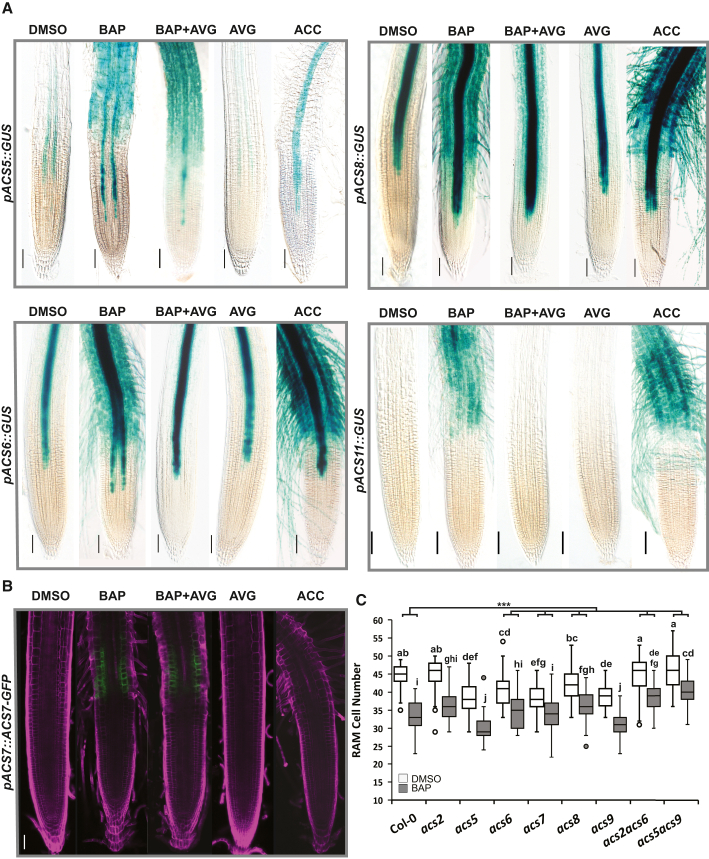
Cytokinin induces the expression of several *ACC SYNTHASE* genes. **(A and B)** Six-day-old seedlings of *pACSx*::GUS (*ACS5*, *ACS6*, *ACS8*, and *ACS11*) transcriptional reporter lines **(A)** and *pACS7*::ACS7:GFP translational fusion lines **(B)** exposed for 24 h to different hormones (5 μM BAP, 5 μM BAP + 1 μM AVG, 1 μM AVG, 5 μM ACC; control is 0.01% DMSO) in liquid media. Scale bars represent 100 μm. **(C)** Number of RAM cortex cells in 6-day-old *WT Col-0* and *acs* mutant lines (*acs2*, *acs5*, *acs6*, *acs7*, *acs8*, *acs9*, *acs2acs6*, and *acs5acs9*) treated for 24 h with 5 μM BAP (control is 0.01% DMSO). Boxplots represent data from three independent replicates (*n* = 15), and the letters show significance classes determined by a linear mixed model ANOVA followed by Tukey’s post hoc HSD test (see supplemental methods). The line-tree at the top of the graph in **(C)** represents the difference-in-differences (DD) estimation between the BAP-reduced RAM size change in WT Col-0 compared with the change in the different *acs* knockouts; the asterisks denote significance at *p* < 0.001. Scale bars represent 100 μm in **(A)** and 50 μm in **(B)**.

To identify the molecular events that mediate cytokinin-induced ACC synthesis in *Arabidopsis* roots, we assayed the cytokinin response of transcriptional *pACS::GUS* reporters (Tsuchisaka and Theologis, 2004), as well as newly prepared lines carrying *ACS2* (*pACS2::ACS2:GFP*) and *ACS7* (*pACS7::ACS7:GFP*) translational fusions (Figure 2A and 2B; Supplemental Figure 3). Of the eight investigated *ACS* genes, the activities of five were induced by cytokinin treatments. The expression levels of *ACS5*, *ACS6*, *ACS8*, and *ACS11* were enhanced in most cell types in the differentiation/elongation zone, as well as in older parts of the root. Cytokinin treatment also strongly upregulated *ACS5*, *ACS6*, and *ACS8* activity in the vasculature and stele of the root tip, whereas *ACS7* activity was induced specifically in the epidermal and cortical cells of the root transition zone. The remaining genes were cytokinin insensitive (*ACS2* and *ACS4*) or were only very weakly activated (*ACS9*; Supplemental Figure 3). *ACS7* was alone among the cytokinin-induced ACSs in responding specifically to cytokinins, as revealed by comparing cytokinin treatment with and without AVG (Figure 2A and 2B). *ACS5*, *ACS6*, *ACS8*, and *ACS11* showed a combination of both cytokinin-induced and cytokinin-induced, ethylene-mediated activation (i.e., activation dependent on ACC production), which was often spatially restricted mostly to the stele/vasculature and non-vascular cell types located proximal to the root transition zone (Figure 2A). In line with the observed cytokinin- and ethylene-responsiveness of several *ACS* genes, we found reduced sensitivity to cytokinin-induced RAM shortening, particularly in *acs6*, *acs7*, and *acs8* single and *acs2acs6* and *acs5acs9* double mutant *Arabidopsis* lines. Moreover, smaller RAMs were also observed under control conditions in single *acs5*, *acs6*, *acs7*, and *acs9* mutant lines compared with WT Col-0 (Figure 2C).

Taken together, our findings imply that cytokinins upregulate ACC production in the *Arabidopsis* root through cell-type-specific transcriptional regulation of several *ACS* genes, using both cytokinin-specific and ethylene-dependent mechanisms. Cytokinin-inducible *ACS5*, *ACS6*, *ACS7*, and *ACS9* regulate RAM size under control conditions and, together with *ACS8*, are necessary for cytokinin-induced RAM shortening.

### *ACO2*, *ACO3*, and *ACO4* are controlled by cytokinins and cytokinin-induced ethylene

**Figure 3:**
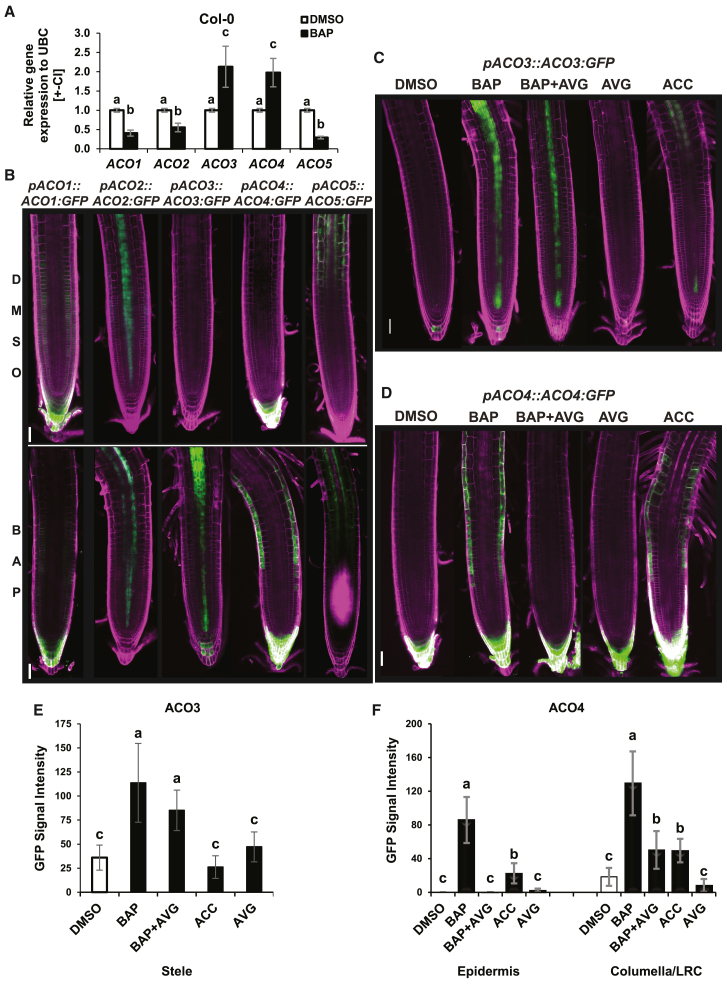
Hormonal control over *ACO3* and *ACO4* genes. **(A)** RT–qPCR quantification of *ACO* (*ACO1–5*) transcripts in 6-day-old *WT Col-0* root tips after 6 h of treatment with 5 μM BAP; 0.01% DMSO served as the control. Relative gene expression is normalized to that of *UBC10* with mean values +/− CI of four biological replicates shown; letters indicate statistically homogenous groups as determined by Kruskal*–*Wallis and Dunn *post hoc* tests. **(B)** Root tips of 6-day-old ACO translational fusions (*pACO1-5::ACO1-5:GFP*) treated for 24 h with 5 μM BAP; 0.01% DMSO served as the control. **(C–F)** Hormonal control of ACO3 **(C)** and ACO4 **(D)** and the corresponding GFP intensities **(E and F)** in the specified tissue files visualized in the root tips of 6-day-old *pACO3::ACO3:GFP* and *pACO4::ACO4:GFP* seedlings, respectively, treated for 24 h with 5 μM BAP, 5 μM BAP + 1 μM AVG, 1 μM AVG, or 5 μM ACC; control is 0.01% DMSO. The areas used for GFP intensity measurements are shown in Supplemental Figure 4. Bars represent the means ± SD; *n* = 10; scale bars in **(B–C)** represent 50 μm.

Our previous findings revealed a possible role for cytokinins as positive regulators of *ACOs* (Zd’arska et al., 2013). Accordingly, we observed that α-aminoisobutyric acid (AIB), an inhibitor of ACO activity (Satoh and Esashi, 1982; 1983), partially rescued cytokinin-induced root shortening (Supplemental Figure 1A and 1B), suggesting a possible role for *ACOs* in cytokinin-regulated root growth. Using RT–qPCR and/or newly prepared reporter lines, we found that exogenously applied cytokinin significantly upregulated *ACO3* and *ACO4* but downregulated *ACO1*, *ACO2*, and *ACO5* in the root tip (Figure 3A and 3B; Supplemental Figure 4). A contrasting effect of cytokinins and ethylene/ACC regulation was observed in the case of *ACO2.* In the epidermis of the transition zone/elongation zone, *ACO2* was downregulated by cytokinins but upregulated by ACC (Supplemental Figure 4A and 4B). Furthermore, cytokinin-induced upregulation of *ACO2* and *ACO3* was observed in the vasculature of the fully differentiated proximal portion of the root (Supplemental Figure 4G and 4H). As with ACSs, we also observed a combinatorial effect of both cytokinin- and ethylene-specific regulation for *ACO3* and *ACO4*. Cytokinin-specific activation of *ACO3* was detected in the stele and the vasculature of the root transition/elongation zone. The *ACO3* activation in the vascular tissues of the more proximal portion of the root (early differentiation zone) turned out to be mediated via cytokinin-induced ethylene production (Figure 3C; Supplemental Figure 4D and 4E). *ACO4* was upregulated in the columella and LRC in both a cytokinin- and an ethylene-specific manner, whereas only ethylene-specific activation was observed in the epidermis of the root transition/early elongation zones (Figure 3D and 3F; Supplemental Figure 4F–4H).

In conclusion, in addition to inducing ACC production, cytokinins are also spatially specific regulators of ACC oxidation, the last step in ethylene biosynthesis. Similarly to their activation of *ACSs*, cytokinins control *ACOs* both directly and via cytokinin-induced ethylene production.

### Multistep phosphorelay and canonical ethylene signaling are necessary for, and cooperate in, cytokinin-induced upregulation of *ACO3* and *ACO4*

**Figure 4:**
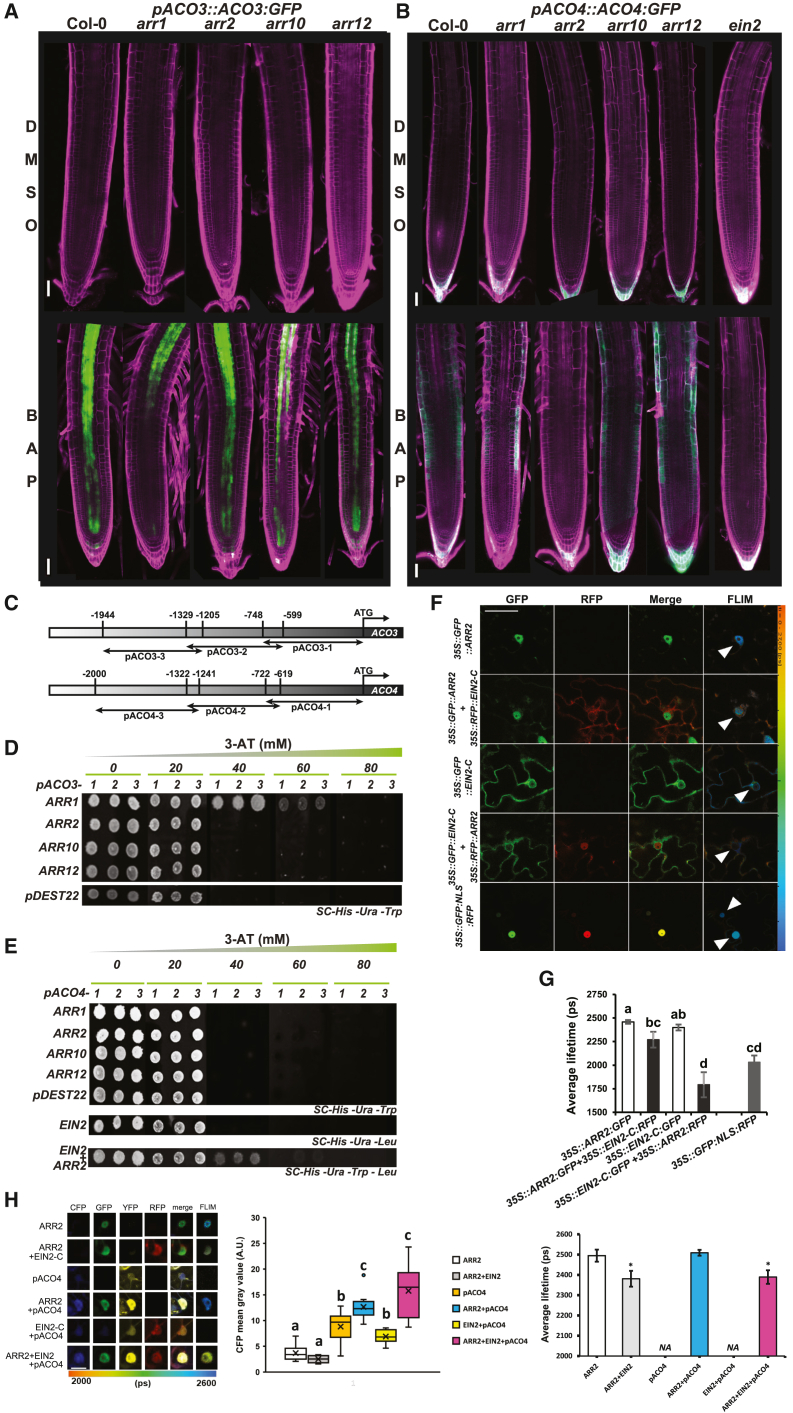
ARR1 directly binds *ACO3*, whereas both ARR2 and EIN2 associate to initiate the transcription of *ACO4*. **(A and B)** *pACO3::ACO3:GFP***(A)** and *pACO4::ACO4:GFP***(B)** in *WT Col-0* and genetic backgrounds deficient in type-B *ARRs* or *EIN2* treated for 24 h with 5 μM BAP; control is 0.01% DMSO. **(C)** Schematic representation of *ACO3* (upper) and *ACO4* (lower) promoter fragments (−1, −2, −3) used in the Y1H assay. **(D and E)** Y1H assays of the binding of truncated type-B ARR TFs (ΔDDKARR1, ΔDDKARR2, ΔDDKARR10, ΔDDKARR12) and pDest22 (negative control) to *ACO3* promoter fragments **(D)** and of truncated type-B ARRs, pDest22, and EIN2-C to *ACO4* promoter fragments **(E)**; the interaction specificity was assayed in the presence of increasing concentrations of 3-amino-1,2,4-triazole (3-AT). **(F and G)** Representative confocal images **(F)** and the fluorescence lifetime **(G)** measured in a FLIM-FRET interaction assay using the indicated vector combinations transiently expressed in *Nicotiana tabacum* leaves; *35S::GFP:NLS:RFP* was the positive control. The white arrowheads indicate FLIM measurement areas. Error bars represent means ± SD of two biological replicates; *n* = 10, and letters indicate statistical significance (two-way ANOVA and Tukey’s *post hoc* test). **(H)** ARR2 and EIN2-C cooperate in the activation of *ACO4*. Representative confocal images (left), CFP mean gray values (middle), and fluorescence lifetimes of tobacco nuclei (right) transiently transformed with combinations of *35S::ARR2:GFP* (full-length *ARR2*), *35S::EIN2:RFP* (*EIN2-C*), and *35S::YFP-NLS-pACO4::CFP-NLS* (*pACO4*) are shown. Mixed model ANOVA and *t*-test, *p* < 0.05; scale bar corresponds to 100 μm. Because the ARR2-GFP lifetime was measured as a donor in the FLIM-FRET assay, no FLIM values were acquired for the combinations without ARR2 (*pACO4* and EIN2) in the FLIM quantification chart (right panel).

To identify the molecular mechanism underlying the cytokinin-induced upregulation of *ACO3* and *ACO4*, *pACO3::ACO3-GFP* and *pACO4::ACO4-GFP* were introduced by crossing into various mutant backgrounds deficient in multistep phosphorelay (*arr1-3*, *arr2-5*, *arr10*-*1*, and *arr12*-*1*) and/or canonical ethylene signaling (*ein2-1*). We found that ARR1 was necessary for cytokinin-mediated upregulation of *ACO3*, whereas both functional ARR2 and EIN2 were required for ethylene-dependent activation of *ACO4* (Figure 4A and 4B). To obtain more detailed mechanistic insight into *ACO* regulation, we assayed the ability of *ACO3* and *ACO4* promoters (*pACO3* and *pACO4*, respectively) to physically interact with RRBs and EIN2-C in a yeast one-hybrid (Y1H) assay. The N-terminal receiver domain of RRBs was previously demonstrated to act as a phosphorylation-dependent negative regulator of the binding of RRBs to DNA (Sakai et al., 2000). Therefore, the interaction of *pACO3/4* fragments was tested with truncated RRB versions (ΔDDKARR1, ΔDDKARR2, ΔDDKARR10, and ΔDDKARR12, Figure 4C; Supplemental Figure 5A) consisting of the C-terminal acidic, GARP DNA-binding/ARRM, and P/Q domains (Sakai et al., 2000; Rieger et al., 2023). Among the tested TFs, only ΔDDKARR1 was able to bind fragments of *pACO3* (Figure 4D). In the Y1H assay, neither ΔDDKARR2 nor EIN2-C were able to bind *pACO4* when expressed separately. However, when co-expressed, ΔDDKARR2 and EIN2-C enabled activation of the yeast reporter under the control of *pACO4* fragments (Figure 4E), suggesting that they bind cooperatively to *ACO4* regulatory sequences. To confirm our findings, we tested the interaction of ARR1 and ARR2 with putative type-B response regulator binding sites that we identified in *pACO3* and *pACO4* (Supplemental Figure 6A and 6B). In a qDPI-ELISA assay (Rieger et al., 2023), we used the GARP DNA-binding domain of ARR1 (G1) and both the GARP DNA-binding domain and entire C-terminal portion of ARR2 (G2 and ΔDDKARR2, respectively, Supplemental Figure 5A; Rieger et al., 2023). We observed sequence-specific interaction of ARR1 (G1) and ARR2 (both G2 and ΔDDKARR2) with oligos selected from *pACO3* and *pACO4*, respectively. However, the interaction of ARR2 (both G2 and ΔDDKARR2) was weaker compared with that of ARR1 (Supplemental Figure 7).

Because EIN2-C does not possess a DNA-binding domain (Zhang et al., 2016), we presume that the ARR2 (ΔDDKARR2) GARP domain might mediate the recruitment of an ARR2/EIN2-C complex to *pACO4*. Accordingly, FLIM-FRET detected a strong interaction of both ΔDDKARR2 and full-length ARR2 with EIN2-C transiently produced in tobacco leaves (Figure 4F and 4G; Supplemental Figure 8A). Compared with ΔDDKARR2, which demonstrated a homogenous nuclear distribution, full-length ARR2 formed nuclear speckles displaying strong interaction with EIN2-C (Supplemental Figure 8A). Weak interaction/ability of EIN2-C to enhance ARR2-mediated transactivation was also detectable in the yeast Y2H assay (Supplemental Figure 8B). To corroborate the functional importance of the ARR2–EIN2-C interaction in the regulation of *ACO4* expression, we used a promoter activation assay based on transient expression in tobacco leaves. In addition to the previously published protocol (Yang et al., 2000), we combined *pACO4*-driven CFP reporter activation with FLIM-FRET-quantified interactions of full-length ARR2 and EIN2-C, transiently overproduced as N-terminal fusions with GFP and RFP (GFP-ARR2 and RFP-EIN2-C), respectively. In contrast to the Y1H assay, GFP-ARR2 was sufficient to upregulate the activity of *pACO4* in the tobacco transient assay. As expected, RFP-EIN2-C alone was not able to activate *pACO4-*driven CFP production. However, when co-expressed, both GFP-ARR2 and RFP-EIN2-C contributed to the enhanced activity of *pACO4* (Figure 4H). Importantly, we observed a statistically significant correlation between the lifetime of GFP-ARR2, reflecting the ARR2–EIN2-C interaction, and the level of *pACO4* activation quantified directly in tobacco cells transiently expressing GFP-ARR2 and RFP-EIN2-C (Figure 4H; Supplemental Figure 8D). This further suggests the functional importance of the ARR2–EIN2-C interaction for the control of *ACO4*.

Taken together, our data indicate that both MSP and canonical ethylene signaling tightly cooperate to bring about the cytokinin-induced upregulation of ethylene biosynthetic genes in the root. On one hand, functional ARR1 is necessary for cytokinin-specific upregulation of *ACO3* in the stele and vasculature of the root transition/elongation zone. On the other hand, ARR2 and EIN2-C interact and mediate cytokinin-induced ethylene-dependent activation of *ACO4* in the root transition zone.

### ACO2, ACO3, and ACO4 are ethylene-synthesizing enzymes involved in cytokinin-induced root and RAM shortening

**Figure 5:**
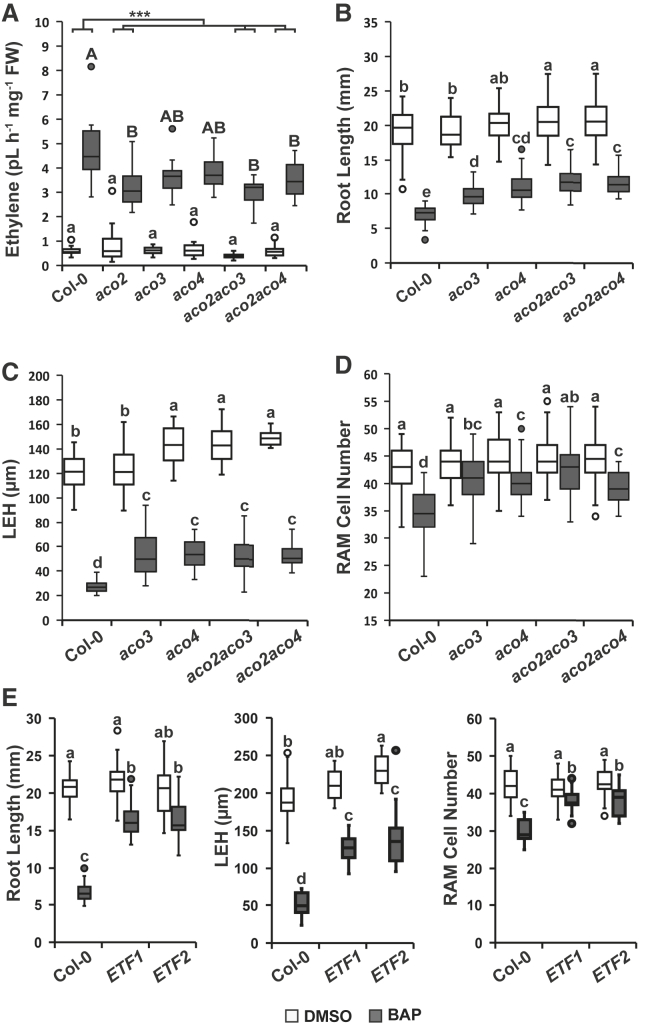
Cytokinin-induced ethylene affects cytokinin-reduced RAM and root elongation. **(A)** Ethylene produced by detached roots (48 h of accumulation) from 6-day-old *WT Col-0*, *aco2*, *aco3*, *aco4*, *aco2aco3*, and *aco2aco4* seedlings treated with 5 μM BAP; control is 0.01% DMSO. **(B–D)** Root length **(B)**, length of the first epidermal cell with a visible root hair bulge (LEH) **(C)**, and RAM cortex cell number **(D)** of 6-day-old *WT Col-0*, *aco2*, *aco3*, *aco4*, *aco2aco3*, and *aco2aco4* seedlings grown on +/−0.1 μM BAP ½ MS; control is 0.01% DMSO. **(E)** Root growth parameters assayed in the presence and absence of cytokinins in ethylene-free (ETF) lines (Li et al., 2022). The boxplots represent data from three independent replicates; *n* = 15; letters above the boxes represent statistically homogeneous groups after a linear mixed model ANOVA followed by Tukey’s *post hoc* test (see supplemental methods). The black line-tree above **(A)** represents difference-in-differences (DD) estimation between BAP-treated WT Col-0 and the different *aco* knockouts; the asterisks denote significance at *p* < 0.001. The white boxes represent mock-treated seedlings, and the gray boxes represent cytokinin treatments.

To assess the functional importance of *ACOs* in cytokinin-induced ethylene biosynthesis and root growth, we measured ethylene formation in roots of the WT and several *aco* mutants in both the presence and absence of cytokinins. Cytokinin treatment strongly upregulated ethylene production in the WT. A statistically significant reduction in ethylene production compared with the WT was detected in cytokinin-treated *aco2* single as well as *aco2aco3* and *aco2aco4* double mutants (Figure 5A). In the presence of cytokinins, the *aco3* and *aco4* single mutant lines showed intermediate ethylene levels, statistically comparable to those of the WT and all ACO2-deficient lines.

We next examined the possible participation of *ACO*s in cytokinin-regulated root growth. Compared with the WT, both *aco2aco3* and *aco2aco4* displayed longer roots under control conditions. A similar trend was also observed in the *aco4* single mutant line, although the difference was not statistically significant (Figure 5B). The inhibition of root length by cytokinin treatment was significantly lower in all tested *aco* mutants compared with the WT. A distinct drop in the sensitivity to cytokinin-mediated root shortening was observed particularly in the *aco4* single and the *aco2aco3* and *aco2aco4* double mutants. A similar response was also seen upon measuring cell elongation (LEH): all the tested mutant lines were less sensitive to cytokinins compared with the WT. Importantly, the *aco4* single mutant and the *aco2aco3* and *aco2aco4* double mutant lines showed extended LEH under control conditions, thus corresponding well with the elongated roots (compare Figure 5C and 5B). In terms of RAM size, the two *ACO3*-deficient lines (*aco3* single and *aco2aco3* double mutants) were either significantly less sensitive or completely resistant to cytokinin-induced RAM shortening, respectively. Although slightly weaker, a similar effect was observed for mutants deficient in *ACO4* (Figure 5D).

To further investigate the possible contributions of the remaining ACOs (ACO1 and ACO5) to cytokinin-regulated root growth, we measured root growth parameters of recently published ethylene-free lines (Li et al., 2022) deficient in all five assayed ACOs (ACO1–ACO5). Compared with all tested single and double *aco* mutants, these lines showed a stronger decrease in sensitivity to cytokinin-induced root and root cell shortening (Figure 5E). However, a similar level of insensitivity was observed in cytokinin-induced RAM size reduction in the ethylene-free lines compared with roots of ACO3-deficient single and double mutants (compare Figure 5D and 5E).

Together, our results demonstrate the involvement of *ACOs* in root growth in either the presence or absence of exogenous cytokinins. *ACO2*, *ACO3*, and *ACO4*, and possibly also *ACO1* and/or *ACO5*, appear to contribute to ethylene-regulated cell elongation and RAM size. Whereas *ACO3* plays a dominant role in the ethylene regulation of RAM size, *ACO4* mediates ethylene control primarily through root cell elongation.

### Working model

**Figure 6:**
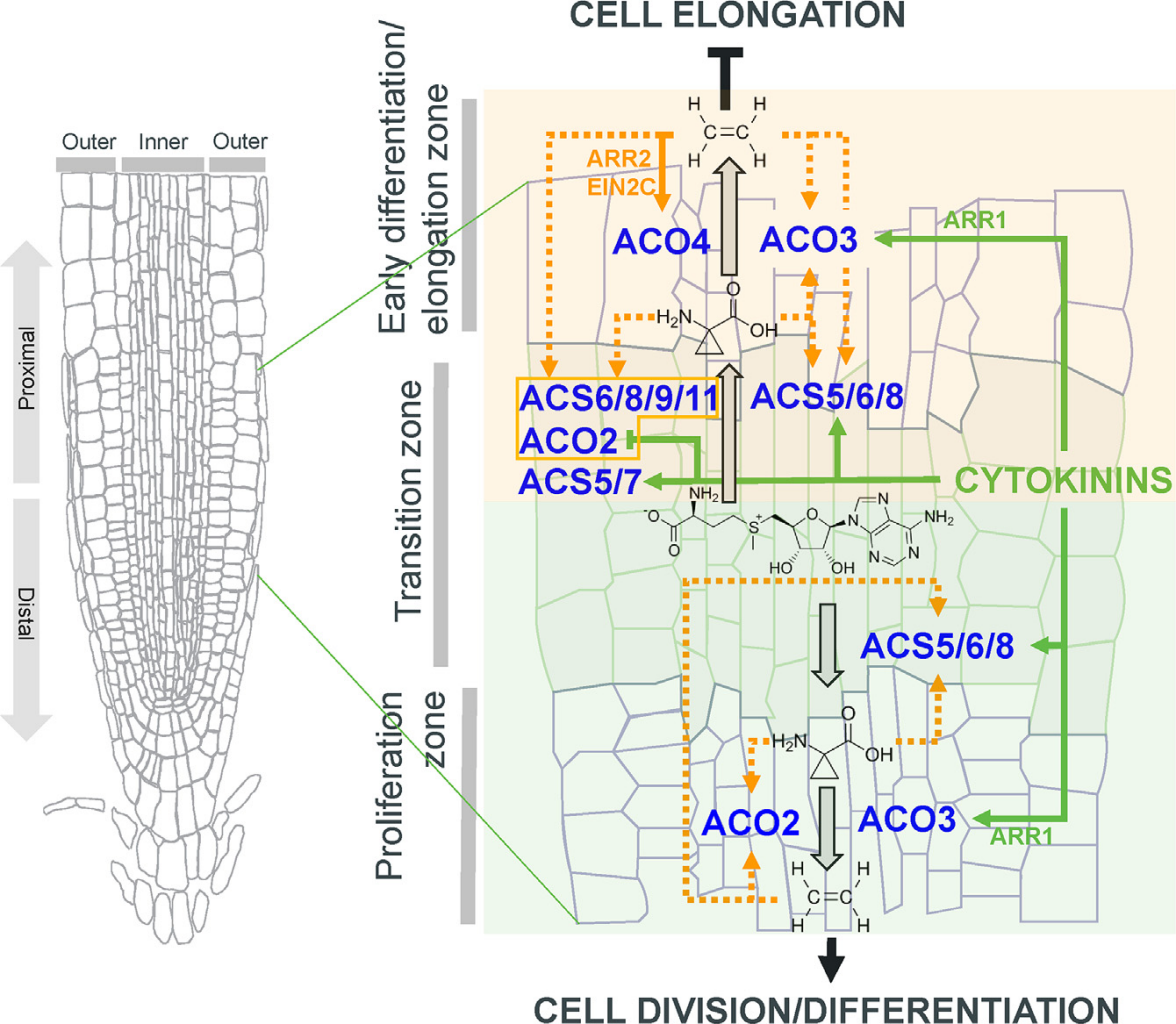
Working model illustrating the role of cytokinin-regulated ethylene biosynthesis in the control of root growth. Cytokinins regulate spatially specific expression of *ACSs* and *ACO* activity to control root growth. Cytokinin- and ethylene/ACC-specific regulations are shown in green and orange, respectively. Dotted lines are used wherever we cannot distinguish between ethylene- and ACC-mediated regulation. *Cytokinin-induced ethylene-mediated* regulation, which leads predominantly to root shortening via inhibition of cell elongation, is highlighted by an orange box, and *cytokinin-regulated ethylene-dependent* regulation, which is associated predominantly with RAM size control via control over the equilibrium between cell division and differentiation in the root transition zone, is highlighted by a green box. See the corresponding portion of the main text for full descriptions. The spatial specificity of individual regulations is depicted only schematically.

On the basis of our data, we conclude that cytokinins regulate ethylene biosynthesis in a cell-type-specific manner to control root growth at the level of both RAM activity and root cell elongation (Figure 6). Cytokinins are able to stimulate both ACC and ethylene production by inducing transcription of several *ACSs* and *ACOs* in a cytokinin-specific as well as an ethylene-specific fashion, which can be spatially traced to the stele/vasculature on the one hand and more peripheral tissue types (epidermis/cortex) on the other. Importantly, ethylene-specific regulation prevails in peripheral and proximal (transition zone and more proximal) tissues. Cytokinin-specific regulation, however, occurs mostly in inner (vasculature/stele) and distal tissues, partially overlapping with the ethylene-specific regulation in proximal tissues but also extending to the QC, as in the case of *ACO3*. This spatial specificity, distinguishable in both longitudinal (proximodistal) and radial axes, appears to be important for cytokinin-induced ethylene-mediated root shortening (which takes place in peripheral and proximal tissues) and cytokinin-induced ethylene-dependent control of RAM size (which prevails in inner and distal cell types). In *cytokinin-induced ethylene-mediated root shortening* (i.e., the response in which the effector is cytokinin-induced ethylene and/or ACC, orange box in Figure 6), cytokinin-induced *ACS5* and *ACS5*/*7* in the stele and epidermis/cortex, respectively, of the root transition/elongation zone enable cytokinin-induced synthesis of ACC. The newly formed ACC might be further metabolized by *ACO3*, which is itself induced by cytokinin-activated ARR1 (possibly at the level of both transcription and phosphorylation, see Supplemental Figure 5B). The resulting ethylene and/or ACC can further stimulate ACC production by upregulating *ACS6/8/9/11* and *ACO2* in the epidermis/cortex and/or *ACS5/6/8* in the vasculature and, through the action of ARR2/EIN2C, also *ACO4*. The ACO2/3/4-mediated ethylene production may be a part of the positive feedback loop involved in ethylene-regulated root growth by attenuating the elongation of cells leaving the RAM. In *cytokinin-regulated ethylene-dependent RAM size control* (i.e., the response that is specific to cytokinins but requires the presence of basal ethylene levels and/or functional ethylene signaling, green box in Figure 6), *ACS6*, *ACS8*, and ARR1-regulated *ACO3* appear to mediate cytokinin-induced ACC/ethylene production more distally in the vasculature/stele of the transition zone/proliferation domain of the RAM. This could be further potentiated by ACC- and/or ethylene-mediated *ACS6/8* and *ACO2* upregulation, eventually leading to the induction of cell differentiation and ethylene-dependent RAM shortening.

## Discussion

### Cytokinins control root growth by regulating the expression of both *ACS* and *ACO*

To date, the only mechanism known to underlie cytokinin-induced ethylene production in *Arabidopsis* has been the cytokinin-induced posttranscriptional stabilization of ACS proteins, specifically ACS2, ACS5, and ACS9 (Vogel et al., 1998; Chae et al., 2003; Hansen et al., 2009; Lee et al., 2017). ACS stabilization does not appear to be specific to cytokinins and can also be mediated by other hormones (Lee et al., 2017; Lee and Yoon, 2018). Nonetheless, this hormone-mediated ACS stabilization has seemed thus far to be limited to etiolated seedlings (see the references above). Here, we show that cytokinins upregulate activity of several *ACS* genes, leading to their transcript accumulation in light-grown *Arabidopsis* roots, as also demonstrated in rice, tobacco, and tomato (Zhang et al., 2009; Zou et al., 2018). We further show that in addition to upregulating ACC production, cytokinins also control the last step of ethylene biosynthesis by differentially regulating the expression of *ACO* genes, controlling cell elongation and RAM size.

In *Arabidopsis*, *ACOs* are members of the large 2-oxoglutarate-dependent dioxygenase (2OGD) superfamily of non-heme iron-containing proteins that have diverse functions. On the basis of amino acid similarity, however, only five of the *ACOs* were proposed to be functional ACC oxidases in *Arabidopsis* (Clouse and Carraro, 2014; Sun et al., 2017; Houben and Van de Poel, 2019 and references therein). Recently, CRISPR/Cas-9-generated quintuple *aco1*,*2*,*3*,*4*,*5* mutant lines were obtained, and the mutants were confirmed to be ethylene-free by gas chromatography (Li et al., 2022). This is in line with our direct ethylene measurements, suggesting that *ACO2*, *ACO3*, and *ACO4* are functional ACC oxidases that mediate ethylene biosynthesis in the root response to cytokinins. ACO2 appears to play a dominant role in ethylene production in the entire root. This is in agreement with the previously identified non-transcriptional upregulation of ACO2 in the cytokinin-treated *Arabidopsis* root (Zd’arska et al., 2013) and possibly also reflects the fact that ACO2 is the most abundant ACO (Supplemental Figure 9; Brady et al., 2007) and is active throughout the differentiated root vasculature.

### Spatial and functional specificity of cytokinin-induced ethylene production

Our data show spatial specificity of cytokinin action in cytokinin-induced ethylene production and the consequent control of root growth. Although increased production of cytokinins in the peripheral tissues located proximally to the root transition zone resulting in high ACC accumulation was associated with strong root-size reduction, upregulation of cytokinin biosynthesis in the vasculature of the more distally located proliferation zone resulted in only a moderate ACC increase with a negligible effect on root length. This is in line with our findings, in which most of the *ACS* genes were upregulated by cytokinins in either the transition zone of the root or more proximally, combining both cytokinin-specific and cytokinin-induced ethylene-mediated regulation. *ACO2* and *ACO4* appear to be important particularly in the latter, i.e., in a putative ethylene-mediated positive feedback loop that upregulates several *ACSs*, and it may act as a mechanism to enhance the effect of cytokinins on ACC and/or ethylene production in elongating root cells. A similar mechanism (positive feedback regulation including ethylene-induced stabilization of ACS2 and ACS6) has been described under stress conditions (Vandenbussche et al., 2012). By contrast, there are fewer ACSs under putative ACC- and/or ethylene-mediated positive feedback regulation in the distal RAM proliferation zone, possibly explaining the lower amount of ACC observed by *IPT* upregulation in the stele/vasculature-specific J2351 activator line. Nonetheless, even the (probably lower) amount of ethylene produced by ACO2 and ACO3 appears to be required for RAM sensitivity to cytokinins, as clearly demonstrated by the nearly complete resistance of the *aco2aco3* double mutant to cytokinin-induced RAM reduction. This was also observed at the level of ethylene signaling, as mutant lines with ethylene-insensitive *etr1-1* mutations as well as *etr1-9 ers1-3* complemented with HK-inactive ETR1 (ETR1-H/G2; Hall et al., 2012) were resistant and/or less sensitive, respectively, to cytokinin-induced RAM shortening (Street et al., 2015; Zdarska et al., 2019). Here, we confirmed this phenomenon by identifying *ACO2/3* as necessary for cytokinin-regulated RAM size. One of the possible mechanisms could be the previously identified ethylene-regulated expression of the type-B response regulators *ARR1* (this work) and/or *ARR10* (Zdarska et al., 2019). Notably, we observed both cytokinin- and ethylene-regulated *ACO4* activity not only in the epidermis of the transition and elongation zone but also in the columella/LRC (Figure 3D–3F; Supplemental Figure 4F). This corresponds well with previous findings in which the LRC was identified as a tissue in which cytokinins control RAM size by regulating auxin degradation (Di Mambro et al., 2019). Ethylene exerts its control over root elongation by controlling both auxin biosynthesis and transport (Ruzicka et al., 2007; Swarup et al., 2007; Stepanova et al., 2008; Vaseva et al., 2018; Zemlyanskaya et al., 2018). Thus, it is tempting to speculate that ACO4-produced ethylene in the LRC contributes to (cytokinin-induced) RAM size regulation, possibly via auxin. That possibility, however, remains to be investigated.

The amount of cytokinin-induced ACC/ethylene may not be the only difference associated with position-specific cytokinin effects on root growth. The spatial specificity that we observed for cytokinin-upregulated *ACSs* and *ACOs* also implies the existence of mechanisms involved in the cell-type-specific ethylene response (i.e., root vs. RAM shortening). This might be due to connections to spatially specific signaling circuits (necessarily being different in differentiated elongated cells and proliferating RAM cells), possibly associated with differential ethylene sensitivity and controlling specific gene sets. Exactly this was recently demonstrated in the epidermis and LRC, the tissues that predominantly control ethylene-mediated root and shoot growth (Vaseva et al., 2018). Our results demonstrating reduced cytokinin sensitivity of lines with attenuated ethylene signaling in the epidermis and/or LRC are in line with this scenario. In addition, we cannot exclude cell-type-specific ethylene distribution reflecting the spatially specific expression and localization of ACOs. Considering the gaseous nature of ethylene, this is rather counterintuitive. Nonetheless, oxygen has been implicated as an endogenous diffusible signal involved in formation of a hypoxic niche in the shoot apical meristem (SAM) organizing center that controls SAM meristematic activity by regulating *WUSCHEL (WUS*) transcription (Weits et al., 2019). This implies the existence of mechanisms that enable cell-type-specific gas distribution in plant tissues, as recently demonstrated for tissue-specific regulation of lipid polyester synthesis genes that ensure a microaerophilic environment in *Lotus* nodules (Venado et al., 2022). That ethylene is insoluble in water may contribute to the possibility of local ethylene action. In parallel, our data imply only a limited ability of cytokinins to be transported (either actively or via passive diffusion) within the diverse cell types of the RAM upon the spatially specific upregulation of cytokinin biosynthesis. This is in accordance with several other reports suggesting a paracrine mechanism of cytokinin action (Bohner and Gatz, 2001; Bielach et al., 2012), possibly mediated via the combined action of cell-type-specific cytokinin biosynthesis and degradation (Miyawaki et al., 2004; Waidmann et al., 2019). The unchanged (WT-like) sensitivity to cytokinin-induced inhibition of root growth in lines with inhibited ethylene signaling in distal/internal tissues (i.e., the stele, the proliferation zone of the RAM, or even the cortex of the root elongation zone) suggests that ethylene produced in the more internal tissues does not effectively move/is not effectively transported to the outer cell files, thus again implying a rather paracrine mechanism of ethylene-mediated inhibition of root elongation. However, whether there is a cell-type-specific ethylene distribution in the *Arabidopsis* root and how it is maintained remain to be demonstrated.

### Both MSP and canonical ethylene signaling interact in the control of *ACO4*

The mechanisms that mediate cytokinin/ethylene crosstalk at the signaling level have been described (for a recent review, see Binder, 2020; Skalak et al., 2021). Here, we demonstrate the existence of a previously uncharacterized signaling mechanism based on a direct interaction between ARR2 and EIN2-C, components of MSP and canonical ethylene signaling, respectively. Our data suggest that both ARR2 and EIN2 are necessary for the ethylene-mediated activation of *ACO4* by cytokinins. ARR2 was found to act downstream of ETR1 in ethylene-dependent signal transduction, possibly mediated by ETR1-dependent ARR2 phosphorylation (Hass et al., 2004). Thus, ethylene might upregulate *ACO4* by activating MSP via ARR2 phosphorylation that recruits the nuclear-localized EIN2-C, a result of the activation of canonical ethylene signaling. Alternatively or in addition, the cytokinin-induced phosphorelay may activate ARR2 by phosphorylation.

How the ARR2/EIN2-C complex mediates *ACO4* upregulation is unclear. In canonical ethylene signaling, EIN2-C, which is unable to directly bind DNA, interacts with EIN2 NUCLEAR-ASSOCIATED PROTEIN 1 (ENAP1), leading to acetylation of histone H3 (H3K14 and H3K23). This induces chromatin to switch to the open state in the ENAP1-binding loci, thus facilitating EIN3-regulated transcription (Zhang et al., 2016, 2017; Wang et al., 2017). On this basis, one may speculate that ARR2 targets EIN2-C to MSP-regulated loci, including *ACO4*, enabling transcriptional activation via EIN2-C-regulated histone acetylation. Nevertheless, the mechanism underlying this type of transcriptional activation remains to be clarified.

### Importance and future outlines

Our findings clearly demonstrate a tight interconnection between cytokinin action and ethylene biosynthesis. Our data imply the existence of a complex network that enables cytokinin control over ethylene biosynthesis at the level of both ACC production and ACC oxidation, the two steps dedicated specifically to ethylene biosynthesis (Depaepe and Van Der Straeten, 2020; Pattyn et al., 2021). Cytokinin-induced *ACSs* and *ACOs* show spatial specificity, correlating with the two major roles of ethylene in the control of root growth: regulation of i) cell elongation in the transition/elongation zone and ii) cell division/differentiation in the transition zone/proliferation domain. Our observations also reveal the existence of potential positive feedback regulatory loops, enabling self-potentiation of ACC and ethylene production. Apart from ACS2/6 stabilization by ethylene under stress conditions (Vandenbussche et al., 2012), this type of regulation has been described for ethylene-regulated *ACS* and *ACOs* in the ethylene-induced wilting triggered by pollination in orchids, suggesting that ethylene is not just a switch, but rather a regulatory factor whose presence is required for a longer period of time (Dolan, 1997 and references therein). We found that *ACO3* is a direct target of MSP signaling and described a novel mechanism in which a physical interaction between proteins mediating MSP and canonical ethylene signaling is involved in controlling *ACO4* expression. Considering the previously identified integration of both ethylene and cytokinin signals in MSP signaling, this type of regulation represents another level of complexity and control in cytokinin/ethylene crosstalk. Both hormones were shown to control root growth and adaptation by mediating interaction between intrinsic developmental pathways, regulating root development and patterning very early in embryogenesis (Yamoune et al., 2021) and in response to environmental signals (Skalak et al., 2021). This allows the root not only to adapt to immediate conditions, e.g., water availability or soil compaction, at the level of root growth and architecture (Saucedo et al., 2012; Park et al., 2018; Chang et al., 2019; Waidmann et al., 2019; Waidmann and Kleine-Vehn, 2020; Pandey et al., 2021; Szmitkowska et al., 2021) but also to anticipate future development and capitalize from past experience via hormone-regulated priming to different stresses (Cortleven et al., 2019; Skalak et al., 2021; Kosakivska et al., 2022; Tiwari et al., 2022). A detailed description of the underlying molecular mechanisms is critical for understanding the principles that activate growth or defense responses in plants and for identifying novel breeding targets. This appears to be highly promising, particularly in the era of targeted crop improvement via genome editing approaches.

## Methods

### Plant materials

*Arabidopsis thaliana* ecotype Columbia-0 (Col-0) was used as the WT and is the background of all mutants and reporters used in this study. All T-DNA knockout lines, as well as the *pACS*::GUS promoter fusion lines from Tsuchisaka and Theologis (2004), were ordered from NASC. *aco2* “AT1G62380” (N674747), *aco3* “AT1G12010” (N682580), *aco4* “AT1G05010” (N514965), *acs2* “AT1G01480” (N16564), *acs4* “AT2G22810” (N16566), *acs5-1* “AT5G65800” (N16567), *acs6* “AT4G11280” (N16569), *acs7* “AT4G26200” (N16570), *acs8* “AT4G37770” (N566725), *acs9-1* “AT3G49700” (N16571), *acs5acs9* (N16593), *AmiRacs* (N16651), *arr1-3* “AT3G16857“ (N6971), *arr10-1* “*AT4G31920*” (N6369), *arr12-1* “AT2G25180” (N6978), *pACS4*:GUS (N31381), *pACS5*:GUS (N31382), *pACS6*:GUS (N31383), *pACS8*:GUS (N31385), *pACS9*:GUS (N31386), *pACS11*:GUS (N31387), *acs2-1acs4-1acs5-2acs6-1acs7-1acs9-1amiRacs8acs11* (*acs8x*; Tsuchisaka et al., 2009), and *eto1-1* (Woeste et al., 1999) were procured from the ABRC. The double mutants *aco2aco3* and *aco2aco4* were generated by crossing the corresponding single mutants. *pA14::EBF2*, *pLRC1::EBF2*, *p35S::EBF2*, *pRCH1::EBF2*, *pS2::EBF2*, and *pCOR::GFP-EBF2* were constructed previously (Vaseva et al., 2018).

The ectopic cytokinin-overproducing lines were prepared using the GAL4>>UAS-based two-component activator–reporter system; J2601 and J2351 activators were respectively crossed to the reporter UAS::IPT for ectopic IPT overexpression or to Col-0 for controls. The F1 generation of the crosses was used for analysis, as these plants were sterile. J2601, J2351, and UAS::IPT (Laplaze et al., 2005) lines were kindly provided by Prof. Eva Benkova (Bielach et al., 2012).

All fluorescent reporter lines produced in this study were generated in the Col-0 background by the floral dip method as described by Clough and Bent (1998), and single-copy homozygous T3 lines were selected and used for analysis. The *arrB-pACO3*::ACO3:GFP, *arrB-pACO4*::ACO4:GFP, and *ein2-1-pACO4*::ACO4:GFP lines were generated by crossing the single *arrB* (*arr1-3*, *arr2-5*, *arr10-1*, *arr12-1*) mutants or *ein2-1* (Zdarska et al., 2019) with the generated reporter lines *pACO3*::ACO3:GFP and/or *pACO4*::ACO4:GFP.

### Growth conditions

Seeds were surface sterilized and sown on half-strength Murashige–Skoog (½ MS) medium (Duchefa Biochemie) with 1% (W/V) sucrose and 1% (W/V) plant agar and then stratified in the dark for 2 days at 4°C. Seedlings were grown vertically under long-day conditions (16-h light/8-h dark) at 22°C for the duration of the treatment.

### Cloning

Unless otherwise specified, all cloning was performed using the Gateway system (Invitrogen) following the manufacturer’s instructions. Fragments were isolated from Col-0 genomic DNA/cDNA by PCR amplification using Phusion High-Fidelity DNA Polymerase (NEB). For each step of the cloning, all cloned sequences were verified by colony PCR, plasmid digestion, and sequencing. The primers used are described in Supplemental Table 1.

### Fluorescent reporters

Entry clones of the native promoters (∼2.5 kb upstream of ATG) and/or gene-coding sequence (as one fragment without the stop codon) of *ACS2*, *ACS7*, and/or *ACO1*,*3-5* were cloned into either pFAST-G04 for transcription and/or pFAST-R07 for translation fusions (Shimada et al., 2010). The *pACO2*::ACO2:GFP clone was prepared by replacing the 35S promoter in the p2GWF7.0 vector (Karimi et al., 2007) with the native *ACO2* promoter by Gibson Assembly (NEB) following the manufacturer’s instructions, and the coding sequence was cloned afterward by LR reaction. The generated clones were pACO3/4:GFP:GUS, pACO1-5:ACO1-5:GFP, and pACS2,7:ACS2,7:GFP.

### Y1H assay and FLIM-FRET

DNA bait and prey clones were generated as described by Reece-Hoyes and Walhout (2018a, b). Overlapping bait promoter fragments of the *ACO3* and *ACO4* promoters (Figure 4) were each cloned into pDONR-P4P1r and then into pMW#2 and pMW#3, respectively, to generate (pACO3/4:HIS and pACO3/4:LacZ). For the cDNA prey clones, the entry clones pENTR_ΔDDK-ARRB and pENTR_EIN2-C, generated by cloning truncated type-B ARRs missing the response regulator domains (ΔDDK-ARR1,2,10,12; see Supplemental Figure 4) and EIN2-Cend into pDONR221, were cloned into pDEST22 for ΔDDK-ARRB (AD-ΔDDK-ARRB) and pGADT7 for EIN2-C (AD-EIN2-C).

For the FLIM-FRET assay, pENTR-ΔDDK-ARR2 and pENTR-EIN2-C (from Y1H) were cloned into the pB7WGR2 and pH7WGF2 destination vectors (Karimi et al., 2007), respectively, for overexpression and N-terminal fusions to both GFP and RFP (35S::GFP:ARR2, 35S::RFP:ARR2, 35S::GFP:EIN2-C, and 35S::RFP:EIN2-C). As a positive control, the binary vector 35S::GFP:NLS:RFP was constructed by fusing NLS:RFP to GFP in the pH7WGF2 destination vector (Karimi et al., 2007) via LR reaction; RFP was isolated from pB7WGR2 to generate pENTR-NLS:RFP by BP; the NLS sequence was added as a part of the forward nls:RFP-attB1-F primer.

## Funding

This work was supported by the Ministry of Education, Youth and Sports of the Czech Republic under the projects TANGENC (CZ.02.01.01/00/22_008/0004581) and LUAUS24277. The work was supported by the 10.13039/501100001659German Research Foundation (CRC 1101 project D02) and the 10.13039/100000011Howard Hughes Medical Institute (to E.M.M.). The work of E.Z. and V.D. was supported by the Russian Science Foundation (20-14-00140).

## Author contributions

A.Y., M.Z., and J.H. conceived the research; J.H. secured funding; A.Y., M.Z., T.D., A.R., E.S., K.B., V.M.-R., M.F., B.P., J.S., P.T., L.T., B.P., K.L.N.M., V.D., E.Z., and A.C. performed the research; A.Y., M.Z., T.D., K.B., V.M.-R., J.S., L.B., I.K., M.P., O.N., E.M., K.H., D.V.D.S., E.Z., and J.H. analyzed the data; and A.Y., M.Z., E.M., K.H., D.V.D.S., and J.H. wrote the paper.

## Supplemental information

Document S1. Supplemental Figures S1‒S9, Supplemental Table S1, and Supplemental Methods

Document S2. Article plus supplemental information
