# Supplementary material for: Cytokinins regulate spatially specific ethylene production to control root growth in Arabidopsis
Source: Plant Commun. 2024 Jul 3;5(11):101013. doi: 10.1016/j.xplc.2024.101013 (PMC11589326; doi:10.1016/j.xplc.2024.101013)
Supplement: Document S1. Supplemental Figures S1‒S9, Supplemental Table S1, and Supplemental Methods [file mmc1.pdf]

Plant Communications, Volume *5*

Supplemental information

Cytokinins regulate spatially specific ethylene production to control root growth in *Arabidopsis*

Amel Yamoune, Marketa Zdarska, Thomas Depaepe, Anna Rudolfova, Jan Skalak, Kenneth Wayne Berendzen, Virtudes Mira-Rodado, Michael Fitz, Blanka Pekarova, Katrina Leslie Nicolas Mala, Paul Tarr, Eliska Spackova, Lucia Tomovicova, Barbora Parizkova, Abigail Franczyk, Ingrid Kovacova, Vladislav Dolgikh, Elena Zemlyanskaya, Marketa Pernisova, Ondrej Novak, Elliot Meyerowitz, Klaus Harter, Dominique Van Der Straeten, and Jan Hejatko

Supplemental Information

Supplemental Figures

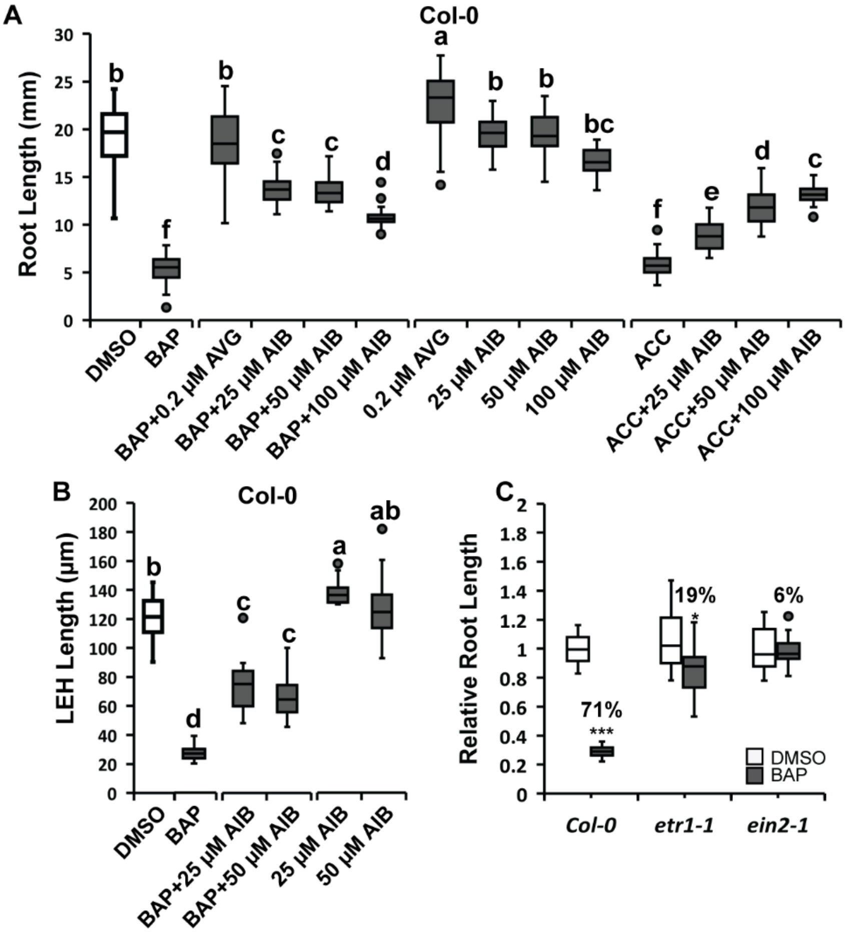

Supplemental Figure 1. Cytokinin-induced root shortening is mediated by ACC/ethylene biosynthesis and ethylene signaling

(**A**) Root length of six-day-old *WT Col-0* seedlings grown on ½MS +/- 0.1 µM BAP combined with AVG or AIB and their respective controls or on +/- 1 µM ACC ½MS with or without AIB. (**B**) Length of the first Epidermal cell with a visible root Hair bulge (LEH) of six-day-old *WT Col-0* seedlings grown on ½MS + 0.1 µM BAP with or without AIB. Boxplots represent data from the three independent replicates, n=20. The letters represent significance classes determined by one-way ANOVA followed by Tukey's post-hoc HSD test. (**C**) Relative root length of six-day-old *WT Col-0*, *etr1-1* and *ein2-1* seedlings grown on 0.1 µM BAP (control is 0.01% DMSO). Boxplots represent root length normalized to the respective mock-treated control (DMSO), n=10, * or *** denote the Student's t-test significance at $p < 0.05$ or $p < 0.001$ respectively.

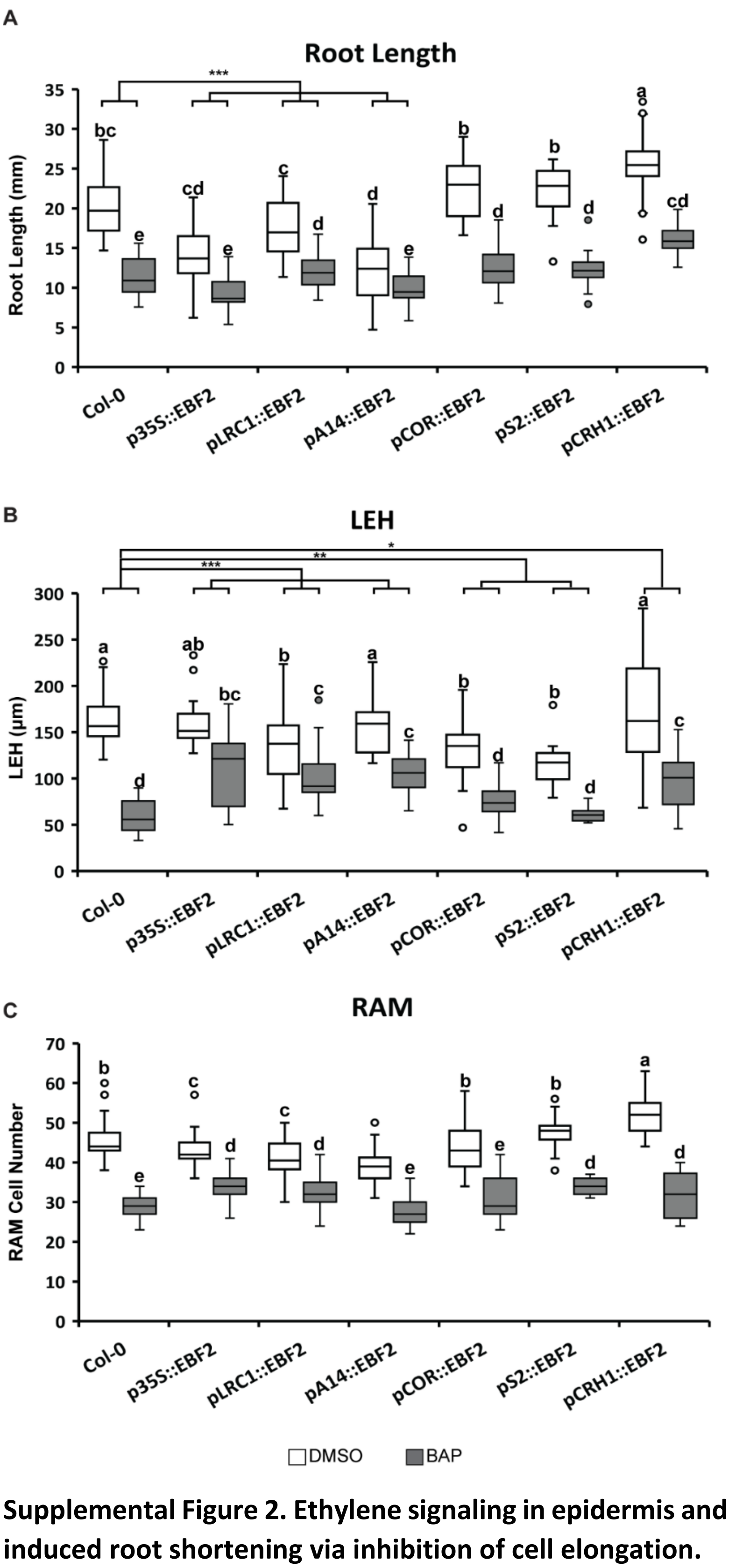

Supplemental Figure 2. Ethylene signaling in epidermis and LRC is necessary for the cytokinin-induced root shortening via inhibition of cell elongation.

(A) Root length, **(B)** differentiated epidermal cell length (LEH) and **(C)** RAM size (cell number) of WT Col-0 (control) compared to lines with cell type-specific inhibition of ethylene signaling via ectopic *EBF2* expression. Following promoters have been used: CaMV 35S (constitutive overexpression in multiple cell types including epidermis), pLRC1 (lateral root cap and epidermis), pA14 (outer cell files of the root including epidermis and lateral root cap), pCOR

(cortex of the TZ/cell elongation zone), pS2 (stele) and pRCH1 (proliferation zone of the RAM) grown on ½ MS media supplemented with 0.1 µM BAP (0.01% DMSO used as control). Boxplots represent data from the two independent replicates, n=15. In (A) and (B), statistically significant changes in the cytokinin sensitivity (differences in differences) between the control and BAP-treated seedlings of individual lines when compared to WT Col-0 are highlighted on the top of each chart.

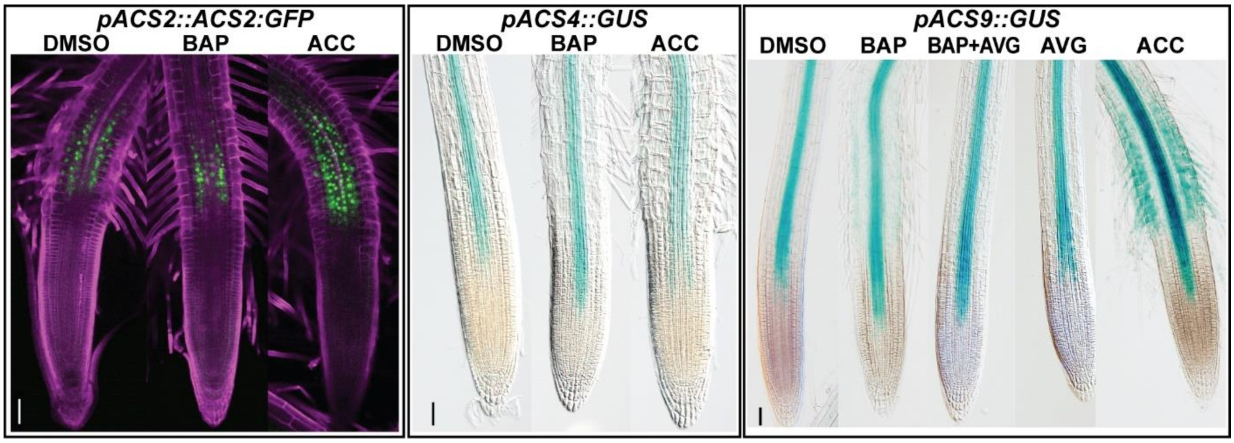

Supplemental Figure 3. Cytokinin non-responsive *ACS* genes

Six-day-old *pACS2::ACS2:GFP*, *pACS4::GUS* and *pACS9::GUS* reporter seedlings treated for 24h with 5 µM BAP, 5 µM BAP + 1 µM AVG, 1 µM AVG, or 5 µM ACC (control is 0.01% DMSO). The scale bars represent 50 µm.

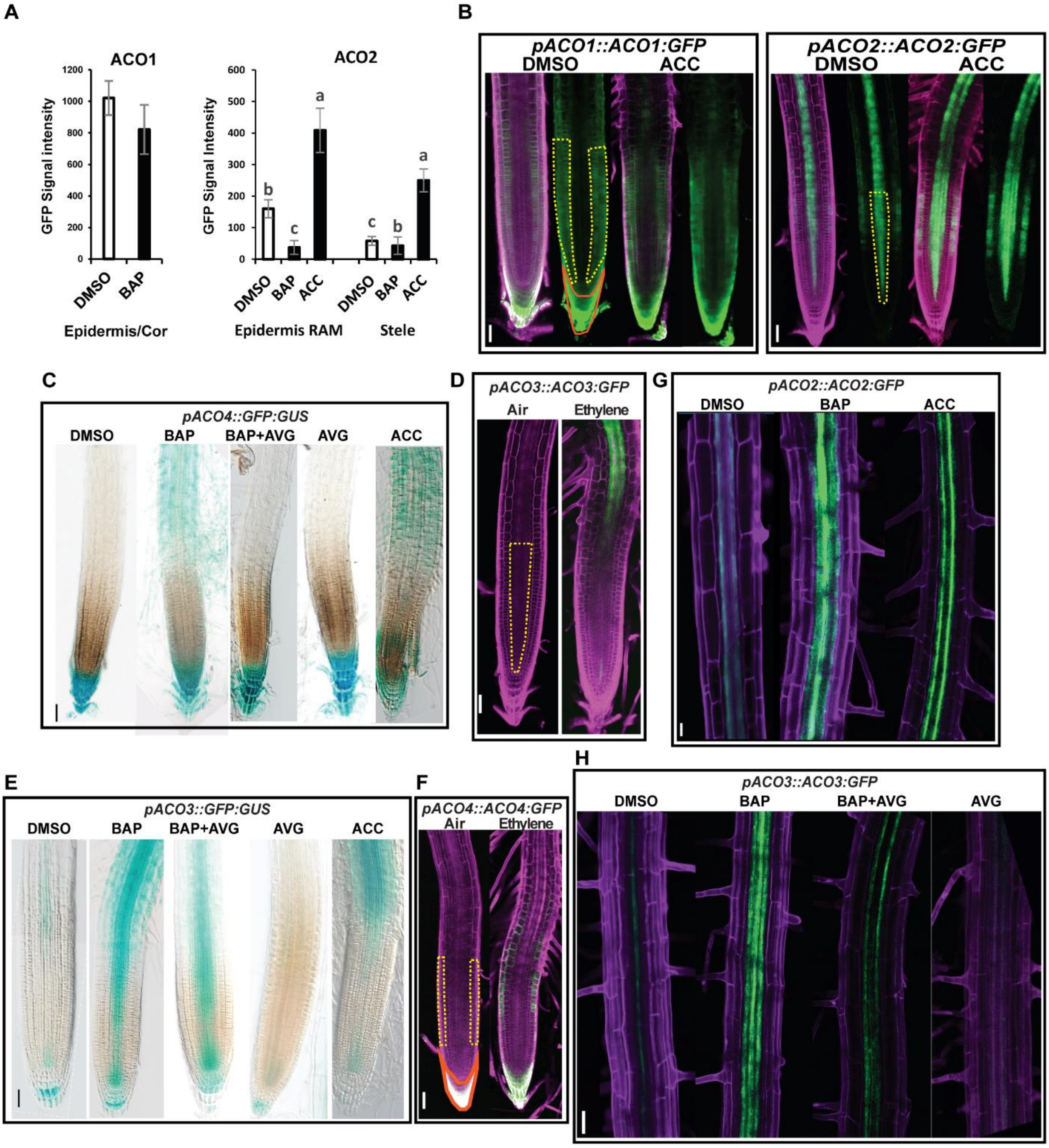

Supplemental Figure 4. *ACO*s are regulated via both cytokinin-specific and cytokinin-induced ethylene-mediated way.

(**A**) Relative GFP signal quantification and (**B**) ACC-mediated induction of *ACO1* and *ACO2* respectively seen in the root tips of six-day-old seedlings of *pACO1::ACO1:GFP* (left) and *pACO2::ACO2:GFP* (right) treated for 24h with 5 µM BAP or 5 µM ACC; control is 0.01% DMSO. Bars represent means ± SD and the letters significance classes (one-way-ANOVA followed by Tukey's post-hoc HDS test). (**C**) Hormonal control of *ACO3* and (**E**) *ACO4,* visualized by GUS staining of six-day-old *pACO3::GFP:GUS* and *pACO4::GFP:GUS* translation fusion lines respectively, treated for 24h with 5 µM BAP, 5 µM BAP+0.2 µM AVG, 0. 2 µM AVG or 5 µM ACC; control is 0.01% DMSO. (**D**) Six-day-old *pACO3::ACO3:GFP* and (**F**) *pACO4*::ACO4:GFP seedlings treated for 24h with 10 ppm of ethylene with air as control. (**G**) Root maturation zone of six-day-old seedlings of *pACO2::ACO2:GFP* and (**H**) *pACO3::ACO3:GFP* treated for 24h with the

indicated hormones (5 µM BAP, 5 µM BAP+0.2 µM AVG, 0.2 µM AVG or 5 µM ACC; control is 0.01% DMSO). The dotted lines in B, D, and F mark the area in which the GFP signal was quantified. The scale bars (B-F, H) represent 50 µm, and 20 µm in (G).

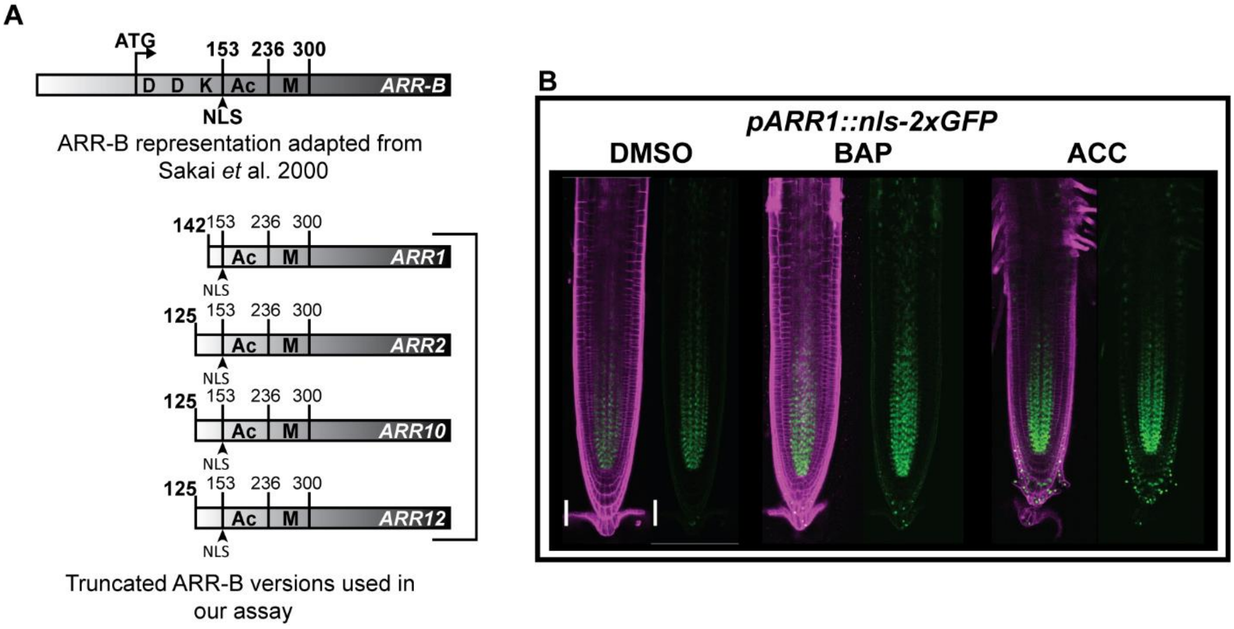

Supplemental Figure 5. Type-B ARRs are under hormonal control and control promoter activity of *ACO3* and *ACO4.*

(A) Schematic representation of the truncated versions of the type-B ARRs [based on (Sakai et al., 2000)] cloned for the Y1H assay. **(B)** Both cytokinins and ACC upregulate *ARR1* in the stele. The scale bars (B) represent 50 µm.

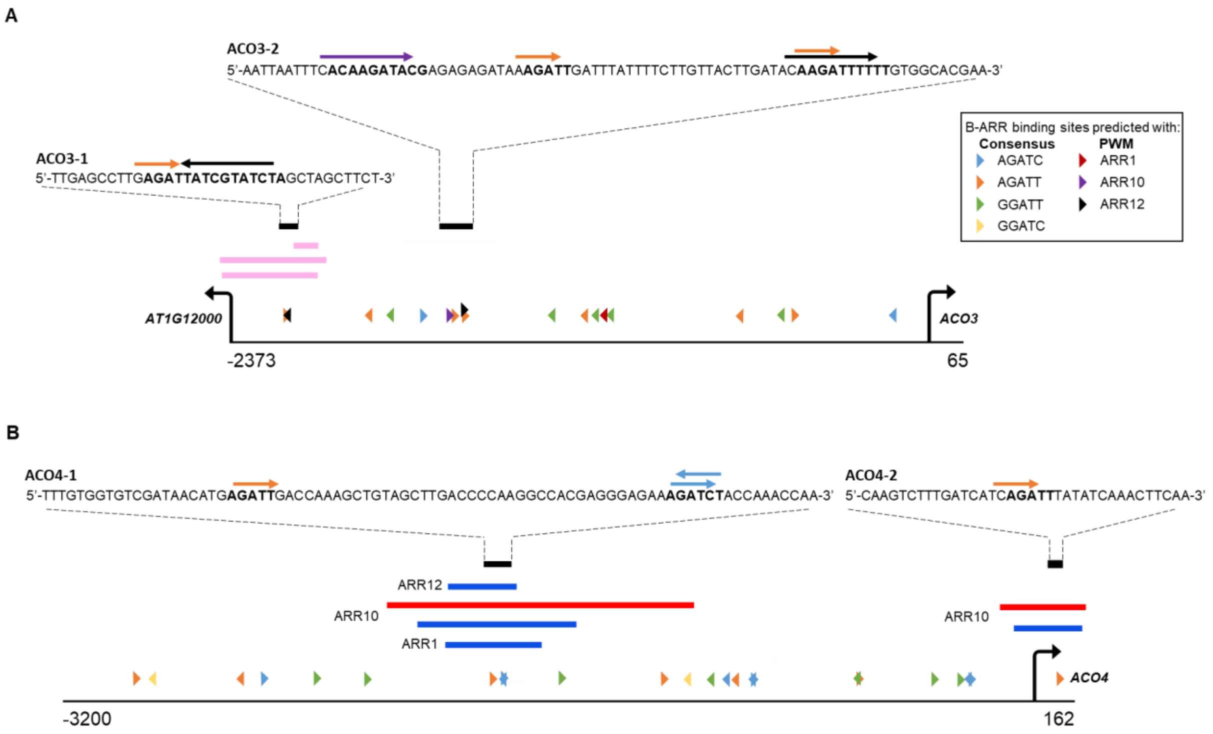

Supplemental Figure 6. Bioinformatic analysis of *ACO3* and *ACO4* promoter regions. The cis-regulatory regions potentially recognized by type-B response regulators identified in the promoter regions of **(A)** *ACO3* and **(B)** *ACO4*. Bold black lines pinpoint the locations of the oligonucleotides used for DPI-ELISA. Bold blue and red lines depict ChIP-seq peaks from Xie et al. (2018) and Zubo et al. (2017), respectively. Bold pink lines designate ATAC-seq peaks from Tannenbaum et al. (2018). Transcription start sites are marked by the curved arrows. Potential binding sites for B-ARRs predicted with either consensus or PWM are represented by colored triangles according to the legend, arrows using the same color code are used to depict the motifs in the DNA sequence of oligonucleotides used for the DPI-ELISA.

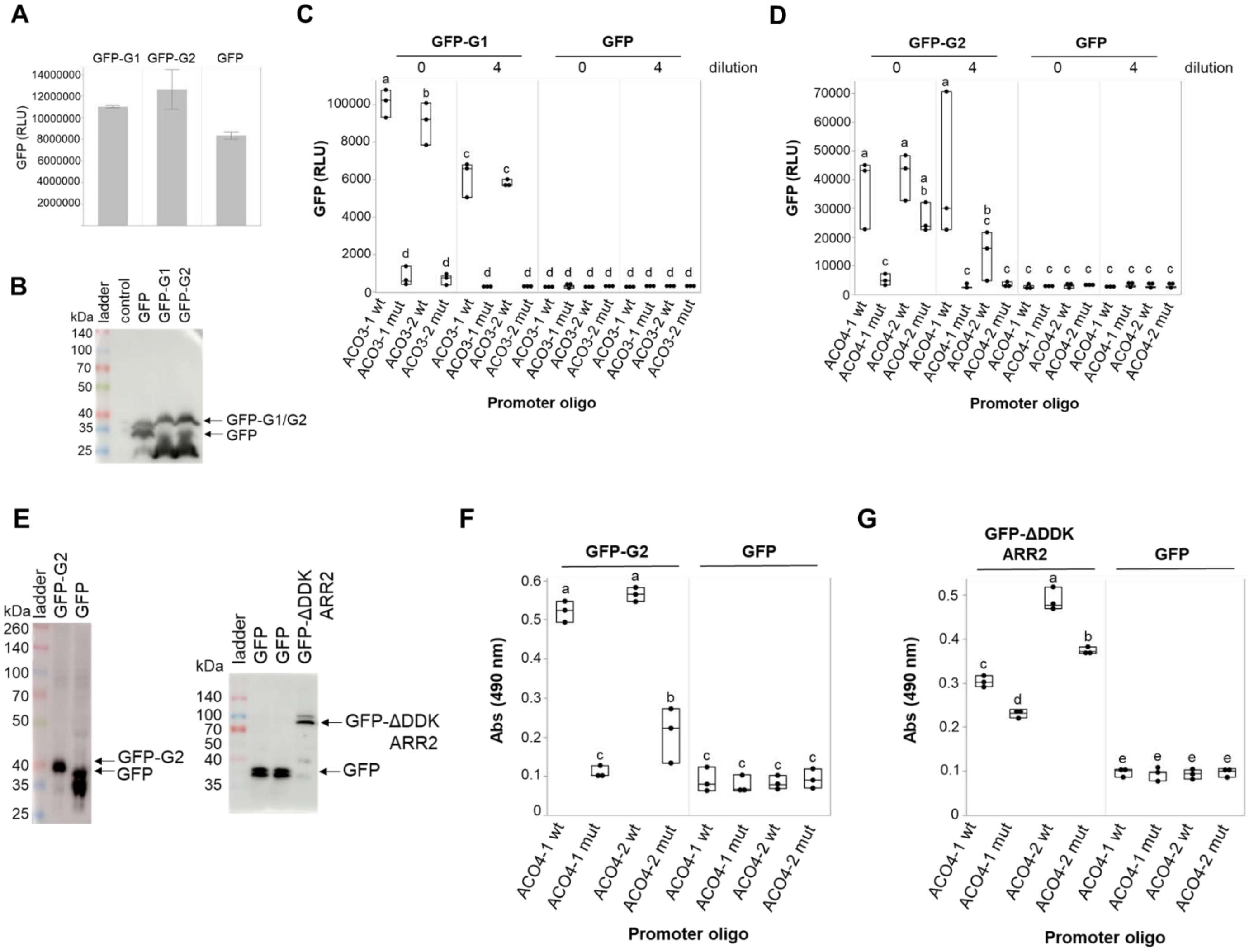

Supplemental Figure 7. ARR1 and ARR2 bind *ACO3* and *ACO4* promoter, respectively. Binding of ARR1 and ARR2 to the fragments of *pACO3* and *pACO4* has been determined using DPI-ELISA assay [see (Rieger et al., 2023) and Materials and Methods]. In brief, biotinylated oligos comprising the putative RRB binding sites identified in the *pACO3* and *pACO4* (see Supplemental Fig. 6) were bound to the streptavidin-coated microtitration plates and incubated with GFP-tagged ARR1 and ARR2 fragments produced in *E. coli*. The GFP-ARR1 and GFP-ARR2 binding has been quantified using fluorescent GFP signal (A-D) or chromogenic detection (E-G) using GFP-specific antibody (α-GFP) and horseradish peroxidase-conjugated secondary antibody (α-mouse-HRP). Oligos with mutated RRB binding sites (mut) and GFP alone have been used as negative controls. **(A)** GFP emission of *E. coli*-produced DNA-binding domain of ARR1/ARR2 (GFP-G1/G2) and GFP amplified at 500V of the null dilutions used in the assay. One-way ANOVA = 0.0629, α=0.05. **(B)** Immunoblot analysis of crude bacterial extracts at null dilutions; control i.e., non-transformed bacteria, α-GFP (1:2,500), α-mouse-HRP (1:10,000). **(C)** Emission of GFP amplified at 600V for GFP-G1 and GFP with *ACO3* oligos after the third washing step. Letters represent significant classes using the LSD test, α=0.01. Levels not connected by a letter are significantly different. **(D)** Emission of GFP amplified at 600V for GFP-G2 and GFP with *ACO4* oligos after the third washing step. Letters represent significant classes using the LSD test, α=0.01. Levels not connected by a letter are significantly different. **(E)** Immunoblot analysis of

crude bacterial extracts at null dilutions; α-GFP (1:2,500), α-mouse-HRP (1:10,000). **(F)** Absorbance at 490nm for GFP-G2 and GFP with *ACO4* oligos. Letters represent significant classes using the LSD test, α=0.01. Levels not connected by a letter are significantly different. **(G)** Absorbance at 490 nm for the fragment of ARR2 comprising entire C-terminal portion (GFP-ΔDDK2 ARR2) and GFP with *ACO4* oligos. Letters represent significant classes using the LSD test, α=0.01. Levels not connected by a letter are significantly different.

A

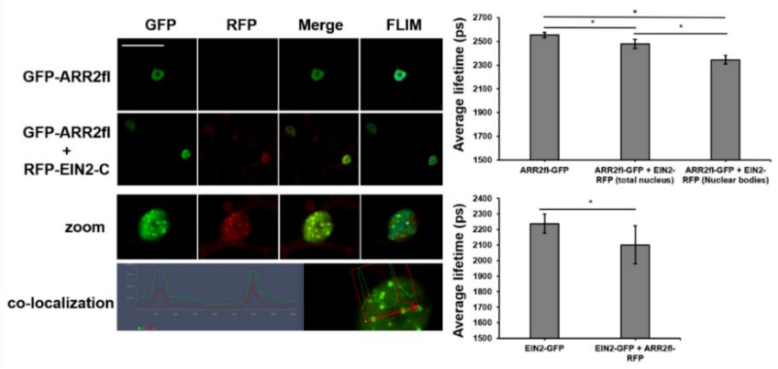
GFP
RFP
Merge
FLIM
GFP-ARR2fl
GFP-ARR2fl + RFP-EIN2-C
zoom
co-localization
Average lifetime (ps)
ARR2fl-GFP
ARR2fl-GFP + EIN2-RFP (total nucleus)
ARR2fl-GFP + EIN2-RFP (Nuclear bodies)
EIN2-GFP
EIN2-GFP + ARR2fl-RFP

B

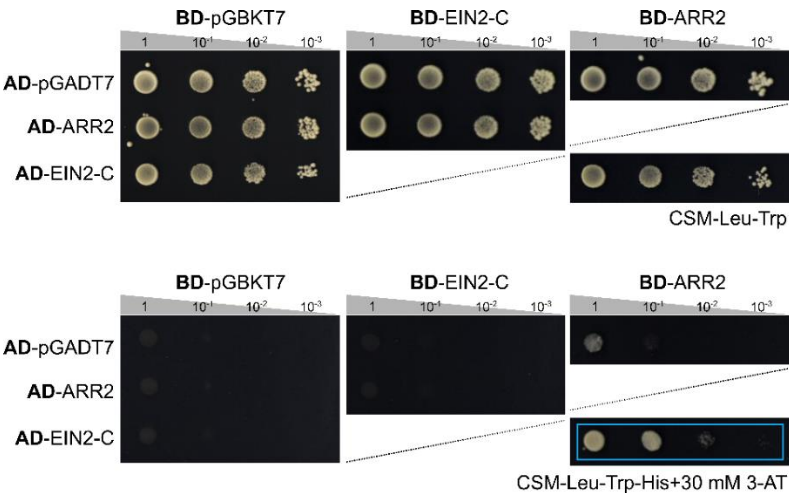
BD-pGBKT7
BD-EIN2-C
BD-ARR2
AD-pGADT7
AD-ARR2
AD-EIN2-C
CSM-Leu-Trp
CSM-Leu-Trp-His+30 mM 3-AT

C

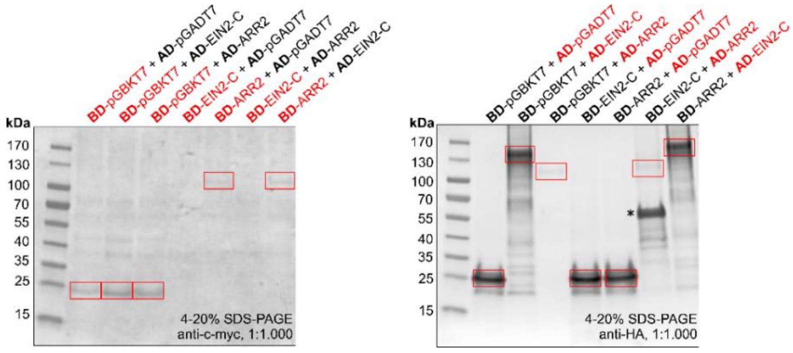
BD-pGBKT7 + AD-pGADT7
BD-pGBKT7 + AD-EIN2-C
BD-pGBKT7 + AD-ARR2
BD-EIN2-C + AD-pGADT7
BD-ARR2 + AD-pGADT7
BD-EIN2-C + AD-ARR2
BD-ARR2 + AD-EIN2-C
kDa
4-20% SDS-PAGE
anti-c-myc, 1:1.000
anti-HA, 1:1.000

D

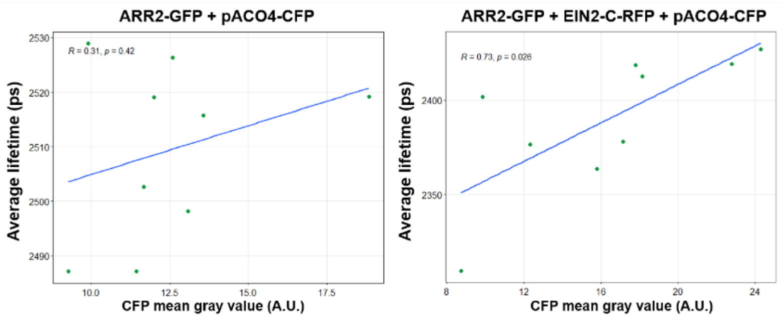
ARR2-GFP + pACO4-CFP
ARR2-GFP + EIN2-C-RFP + pACO4-CFP
R = 0.31, p = 0.42
R = 0.73, p = 0.026
Average lifetime (ps)
CFP mean gray value (A.U.)

Supplemental Figure 8. Full-length ARR2 interacts with EIN2-C.

(A) Representative confocal images and the fluorescence lifetime measured in the FLIM-FRET interaction assay using the indicated vector combinations transiently expressed in *Nicotiana tabacum* leaves. Corresponding line profile shows the level of colocalization between ARR2fl and EIN2-C. Bars represent means ± SD of three biological replicates and asterisks indicate statistical significance (t-test; $P < 0.05$). **(B)** Y2H assay to assess interaction between ARR2 and EIN2-C. Yeast co-transformed with BD- and AD-clones were cultivated for 4 days at 28°C on either vector-selective media (-Leu-Trp) or interaction-selective media (-Leu-Trp-His + 30 mM 3-AT). The empty pGADT7 and pGBKT7 plasmids expressing the AD and BD domains, respectively, were used as negative controls. AD, activation domain; BD, DNA-binding domain. A weak transactivation activity of ARR2 alone is detectable in the case of BD-ARR2, however, yeast growth is clearly upregulated when combined with AD-EIN2-C (highlighted by blue rectangle). No interaction is detectable in the case of swapped domains (BD-EIN2-C + AD-ARR2), most probably due to the absence or undetectable levels of BD-EIN2-C in the system (see panel B). **(C)** Western blot analysis of AD- and BD-fusion proteins extracted from co-transformed yeasts used in the Y2H assay. AD- and BD-fused proteins were identified using anti-HA and anti-c-myc antibodies, respectively. Proteins of the correct size are highlighted with red rectangles, except for BD-EIN2-C, which was not detected in our system. A black asterisk marks a non-specific band detected in BD-EIN2-C + AD-ARR2 co-transformed yeasts using anti-HA antibody. **(D)** Correlation between the strength of the *pACO4*-driven CFP signal intensity and GFP fluorescence lifetime measured either in the presence of GFP-ARR2 only (left) or GFP-ARR2 and RFP-EIN2-C (right). Statistically significant correlation has been observed only in case of presence of both GFP-ARR2 and RFP-EIN2-C (right), suggesting functional importance of the ARR2/EIN2-C interaction for the *pACO4* activity.

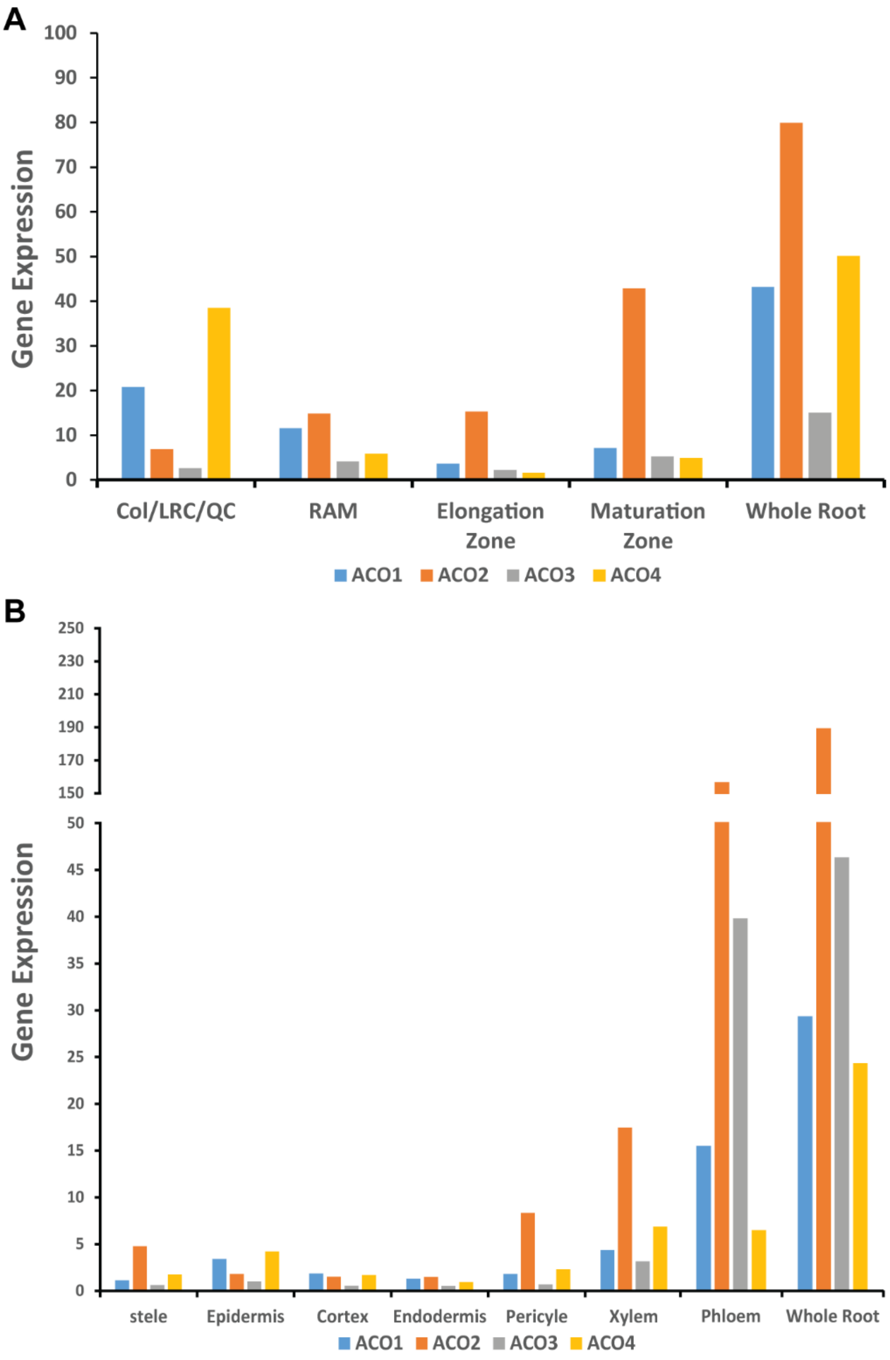

Supplemental Figure 9. *ACO* gene family expression in the *Arabidopsis thaliana* root.

Expression of different ACO genes (*ACO1*-4) in the root of *Arabidopsis thaliana* in the longitudinal **(A)** and the radial **(B)** axes. Data based on Brady et al. (2007) and were extracted from http://bar.utoronto.ca/eplant/. While *ACO2* is rather poorly expressed in the columella/LRC/QC, it is the *ACO* with highest expression levels in the rest of the root.

Supplemental Methods

Hormonal treatments

Depending on the type of analysis we used two types of assays. Direct treatments were used for the root and LEH length, where seeds were sown directly and grown continuously on media containing BAP (B3408, Sigma-Aldrich), ACC (A3903, Sigma-Aldrich), AIB (850993, Sigma-Aldrich), or AVG (32999, Sigma-Aldrich). For the RAM and reporter lines analysis, seeds sown and grown on ½MS, were transferred to liquid ½MS supplemented with the different hormonal treatments.

GUS staining

Six-day-old *pACS::GUS* and *pACO3-4::GFP:GUS* seedlings were stained for GUS expression as described in Malamy and Benfey (1997). Differential Interference Contrast microscopy (Olympus BX61) was used for imaging. 1 mM of Fe salts (K3, K4) were used. *pACS::GUS* seedlings were stained for 15 min and *pACO::GFP:GUS* ones for 30 min .

Root and RAM visualization, measurements and reporter image analysis

Roots were stained with propidium iodide (PI; P4864, Sigma-Aldrich; 50µg per ml of H2O for 7min) and imaged using the inverted confocal microscopy system Zeiss LSM 800 or LSM 880 to visualize root cells. RAM size was determined according to Dello Ioio *et* al. (2007), as the number of the cortex cells counted from the quiescent center to the first elongated cell. The length of the cortical cells was first measured using Cell-O-Tape macro (French et al., 2012) in ImageJ/Fiji software (Schindelin et al., 2012) (http://rsb.info.nih.gov/ij/) and the first elongated cell was designated by performing a point change test of the cells length using "The Multiple Structural Change algorithm" tool available at http://www.ibiologia.com.mx/MSC_analysis (Pacheco-Escobedo et al., 2016). The first epidermal cell with visible root hair bulge (LEH) was determined as described in Le *et* al. (2001). Both LEH and root lengths were measured using ImageJ/Fiji software. For root elongation, plates were scanned with the same ruler to set the scale.

ACC measurement

50 root tips from each sample were cut and frozen in cold water and assessed for ACC levels. ACC quantification was performed using a modified version of the method previously published by (Salazar et al., 2012). Briefly, roots of *Arabidopsis thaliana* were extracted into 1ml of H_2O:methanol (1:1) extraction solution and d_4-ACC was added as an internal standard. The samples were homogenized using a bead mill (MixerMill, Retsch GmbH, Haan, Germany) and then centrifuged for 15 minutes at 18 000 rpm at 4°C. 400 µL of supernatant was evaporated

to dryness and derivatized with the AccQ-Tag Ultra kit (Waters, Milford, MA, USA). For ACC quantification, the samples were analyzed by liquid chromatography–tandem mass spectrometry (LC-MS/MS) in multi reaction monitoring (MRM) mode employing the LC-MS/MS system 1260 Infinity II LC System coupled to a 6495 Triple Quad LC/MS System with a Jet Stream and Dual Ion Funnel technologies (Agilent Technologies, Santa Clara, CA, USA).

Quantification of reporter gene expression

Quantification of the pACO1-4::ACO1-4:GFP signal was performed in Fiji as described in Zdarska & Cuyacot (2019), the region of interest for each gene was defined as in supplemental figure 3.

In vivo ethylene measurement

Six-day-old Col-0, *aco2*, *aco3*, *aco4*, *aco2aco3,* and *aco2aco4* seedlings were grown vertically on a mesh on ½MS (1% (w/v) sucrose solidified with 0.8% (w/v) agar. The roots were then cut and wound ethylene was allowed to dissipate for 4h on the plate (to preserve humidity). Then 30 roots from each of the tested genotypes were subsequently transferred to 10 mL chromatography vials (Chromacol, VWR) with 4 mL liquid ½MS + 5 µM BAP or 0.01% DMSO as a control. The vials were hermetically sealed with rubber stops and snap-caps (Chromacol, VWR). Roots were incubated for 48h with gentle shaking to allow ethylene accumulation in the headspace. Ethylene emanation was measured using laser-based photoacoustic spectroscopy (ETD-300, Sensor Sense, The Netherlands) according to Van de Poel & Van Der Straeten (2017). Three biological replicates were measured; five vials containing 30 seedlings each, were used for each biological replicate and each treatment/genotype combination. 4 blank vials, containing only medium were used as controls for the measurements. Data were normalized to the blank control and expressed per hour per mg fresh weight.

Yeast transformation, mating and Y1H screening

The DNA-bait clones were integrated into YM4271 yeast strain genome and the AD-TF transformed into Y187α as described by Reece-Hoyes and Walhout (2012). AD-ARR2 and AD-EIN2-C were co-transformed as well. To screen AD-TF/bait-HIS3 activation, mating was allowed as described in Castrillo et al. (2011). The diploid-selective-media used were: CSM-His-Ura-Trp for AD::ARRs/bait-HIS3; CSM-His-Ura-Leu for EIN2-C/bait-HIS3, and CSM-His-Ura-Trp-Leu for AD::ARR2+AD::EIN2-C/bait-HIS3. The screening plates contained the diploid-selection-media ± an ascending concentration of 3-AT (0 mM, 20 mM, 40 mM, 60 mM, and 80 mM). The YM4271 and Y172α yeast strains were kindly provided by Helene Robert Boisivon, Ph.D..

Yeast two-hybrid assay

The full length ARR2 was isolated from the WT cDNA and cloned into the pDONR221 via BP reaction to generate the entry clone. Both ARR2 and the EIN2-C entry clones were each, respectively, cloned into pGADT7 and pGBKT7 (Horak et al., 2008) via LR reaction to generate the expression clones AD-ARR2, AD-EIN2-C, BD-ARR2, and BD-EIN2-C.

Destination vectors pGBKT7 and pGADT7 were derived from the Matchmaker^{TM} System (Clontech). Transformation of yeast strain PJ69-4A and growth assays were performed as described previously (Horak *et al.*, 2008). The yeast growth was tested on vector- (CSM-Leu-Trp) and interaction-selective media (CSM-Leu-Trp-Ade, CSM-Leu-Trp-His, CSM-Leu-Trp-His + 3-30 mM 3-amino-1,2,4-triazole (3-AT)).

The expression of all analyzed proteins in transformed yeasts was further confirmed by Western blot analysis. Briefly, yeast colonies were grown in a vector-selective medium (CMS-Leu-Trp) to reach the late log phase (OD_{600} > 1.5). Samples of yeast cultures were centrifuged, and pellets were lysed with glass beads and lysis buffer (8 M urea, 4% SDS, 50 mM Tris/HCl pH 6.8, 0.1 M DTT, 30% glycerol, and 0.005% bromophenol blue). Protein samples were separated by SDS-PAGE (4-20% gradient gel) and then transferred to polyvinylidene difluoride membranes. Membranes were subjected to Ponceau S staining and immunoblot analysis as described previously (Zdarska et al., 2019).

FLIM-FRET

Plasmid vectors (*35S::GFP:ARR2, 35S::RFP:ARR2, 35S::GFP:EIN2-C*, and *35S::RFP:EIN2-C* as well as the positive control *35S::GFP-RFP*) were transiently expressed in *Nicotiana tabacum* (SR1 Petit Havana) epidermal leaf cells using the infiltration procedure described in (Voinnet et al., 2000). Gene silencing in *Nicotiana tabacum* was suppressed by co-infiltrating the p19 protein from tomato bushy stunt virus cloned into pBIN61 (Voinnet *et al.*, 2000). The Zeiss LSM 780 Axio-Observer laser scanning confocal imaging microscope equipped with external In Tune laser (488-640 nm, < 3nm width, pulsed at 40 MHz, 1.5 mW) C-Apochromat 63 x water objective, NA 1.2 and the HPM-100-40 Hybrid Detector from Becker and Hickl GmbH was used for FLIM-FRET data acquisition. FLIM analysis was performed using a Simple-Tau 150N (Compact TCSPC system based on SPC-150N) with DCC-100 detector controller for photon counting. For GFP and RFP excitation, we used the Tune laser at 490 nm wavelength and a DPSS-laser at 561 nm, respectively. Zen 2.3 light version from Zeiss was used for processing confocal images. SPCM 64 version 9.8 was used to acquire FLIM data and SPCImage version 7.3 from Becker and Hickl GmbH for data analysis. For each analysis, the nuclear area was selected as region of interest containing signals for fluorescent lifetime calculation. A multiexponential decay model was used for fitting. Lifetime components with very low values below 500 ns were considered as background and avoided for average lifetime calculations.

Gene expression analyses

The root tips from 6-DAG seedlings treated for 6 hours (5µM BAP with 0.01% DMSO as a control) were cut off with a scalpel and immediately frozen in liquid nitrogen. Total RNA from the collected tissue was isolated using the RNAqueous Small Scale Phenol-Free Total RNA Isolation Kit (Ambion) according to the manufacturer's instructions. cDNA was prepared using RTP3 primer and Superscript III (Invitrogen) according to the manufacturer's instructions, and RT–qPCR was performed using the FastStart SYBR Green Master Kit (Roche) according to the manufacturer's instructions on a Rotor-Gene 6000 (CORBETT RESEARCH) instrument. RTP3 primer: 5'-CGTTCGACGGTACCTACGTTTTTTTTTTTTTTTTTT-3'. Four independent replicas were processed. For relative quantification of *ACO1-5* transcripts, we used the primers in table 2 (*ACO3* and *ACO5* as described in (Schellingen et al., 2014) and *ACO1-2* and *ACO4* as described in (Lee et al., 2017). For individual *ACOs* pairs of primers sequences are in the Supplemental material table 1. Average relative quantities were normalized to internal controls *UBIQUITIN-CONJUGATING ENZYME 10* (*UBC10*) and to mock-treated controls that correspond to value 1 (qbase+, Biogazelle). For statistical analysis, a Mann–Whitney test was performed (qbase+, Biogazelle).

Transient promoter activation assay

The construction of the *35S::YFP-NLS-pACO4::CFP-NLS* (designated as *pACO4-CFP*) gene construct involved a combination of Gateway and restriction cloning methodologies. *pACO4* promoter sequence (2000 bp) was fused with the *CFP-NLS* through the PCR using primers with attB overhangs and pK7CWG2 vector. The final construct assembly was achieved by restriction-based cloning, where the YFP-NLS marker with stop codon was incorporated between 35S promoter and *pACO4::CFP-NLS* sequence resulting in final *35S::YFP-NLS-pACO4::CFP-NLS* vector. Detailed primer sequences are provided in the supplementary materials. CFP intensity was measured as a mean gray value. For confocal scanning, we used lasers 409 nm, 490 nm, 514 nm, and 561 nm to simultaneously scan CFP, GFP, YFP, and RFP respectively. Due to the significant spectral overlap between GFP and YFP emission spectra, distinguishing between the two fluorophores during confocal microscopy proved challenging (additive effect on YFP fluorescence intensity in combination with GFP, and vice versa). To confirm the specificity of fluorescence signals attributed to individual fluorophores, fluorescence intensity was assessed by switching off the corresponding laser excitation, ensuring the signal observed was indeed originating from the targeted fluorophore.

DPI-ELISA

Two potential CRM binding regions for each promoter were chosen and synthesized, with one oligo for each double strand pair being labelled with biotin at the 5' end (see Supplemental Tab. 1). DNA binding domain of ARR2 (G2) named GARP2 in *pET-Dest42-GFP* is described in (Rieger *et al.*, 2023). DNA binding domain of ARR1 (G1) was cloned into *pET-Dest42-GFP* via Gateway®

swapping. Truncated fragment of ARR2 missing the N-terminal receiver domain (ΔDDK2-ARR2) was cloned into *pET-Dest42-GFP* via Gateway® swapping. All primers are listed in the Supplemental Table 1. BL21(DE3)-RIL (Agilent) bacteria were grown and proteins and extracted as in (Rieger *et al.*, 2023) with the following changes: pre-cultures were grown in Luria-Bertani medium overnight at 37°C; 1 mL was used to inoculate 300 mL Terrific Broth medium and grown at 37°C to about OD_{600} 0.4, cooled on ice, upon which 1mM IPTG was added for protein induction and cultures grown for 6hrs at 25°C or 16hrs at 18°C. After clarification for experiments, the proteins were diluted 1:1 with DPI-Ex-buffer to make the initial dilution.

The GFP-based ELISA was performed as in (Rieger *et al.*, 2023) using Pierce® Steptavidin High Binding Capacity Coated 384-well black plates (Thermo Scientific) with the following changes: final total sample volume was 45µL, PMT voltage given in figure legends, measured after each wash step, alternating buffers as described. The chromogenic-based ELISA was performed essentially in (Brand et al., 2010) using Pierce® NeutrAvidin® Coated 96-well plates (Thermo Scientific) with the following changes: TBS-T 0.05% Tween-20; all wash steps with 200µL TBS-T, and all protein and antibody incubation steps in 100µL; OPD reaction was 5mg OPD substrate in Sodium-Citrate buffer following the manufacturer's instructions (product: 34006, Thermo Scientific). Antibodies α-GFP (mouse-IgG_1 κ, Roche), α-mouse-HRP (goat IgG, Sigma) were used for the ELISA and western blotting. Absorbance was measured on a TECAN Safire at 490nm (stopped reaction) by adding 50µL 2.5M sulfuric acid. Immunoblotting protein marker Spectra™ Multicolor Broad Range Protein Ladder (Thermo Fischer Scientific). Chemiluminescence was detected using ECL Select™ (cytiva, Amersham™) on ImageQunat 800 (Amersham).

Statistics and graphing were done using JMP16® (SAS) using one-way ANOVA and the Fischers Least Squared Difference test, with the alpha levels indicated in the figure legends.

Statistics

All charts (unless otherwise mentioned) were drawn using Microsoft Excel 365®. The statistical analysis, pairwise comparisons, post hoc tests were performed in RStudio. For RAMs, LEH lengths, root lengths, ethylene measurements data were treated as follows: the outliers were detected using the boxplot method, where the data were divided into Genotype and Treatment condition sub-sets. For each subset values above Q3[1] + 3xIQR[2] or below Q1 - 3xIQR were considered extreme points and were removed before further analysis. The effect of different treatments and genotypes on ethylene production was assessed by fitting a linear mixed effect model via restricted maximum likelihood. The experiment was carried out with 2 or 3 biological replicates which was introduced as a random effect in the model to account for intra-cluster correlation between the responses from one replicate. The model was defined considering the interaction between genotype and treatments. Pairwise comparison was carried out using Kenward-Roger degrees-of-freedom calculation and Tukey's adjustment. For ethylene

[1] 3rd quartile
[2] inter quartile range

measurements, the pairwise comparison of genotype was calculated independently for each treatment group, as the result of the mixed model stated the differences between treatments clearly ($p<0.001$). For all the other measured characteristics pairwise comparisons were computed among genotypes and treatments together. In case of Figure 4H, false discovery rate multiple testing correction has been used. The *lmer* and *emmeans*[3] packages of software R were used to calculate the results (Bates et al., 2015; R_Core_Team, 2021).

[3] Lenth, Russell V., Paul Buerkner, Maxime Herve, Maarten Jung, Jonathon Love, Fernando Miguez, Hannes Riebl, a Henrik Singmann. “emmeans: Estimated Marginal Means, aka Least-Squares Means”, 08. september 2022. https://CRAN.R-project.org/package=emmeans

Supplemental Table 1: List of primers used.

Gene	PRIMER	SEQUENCE	AIM
**ACO1**	pACO1-attB1-F	GGGGACAAGTTTGTACAAAAAAGCAGGCTTAccggtggttggagaacgaga	BP: pDONR221 then
	ACO1-attB2-R	GGGGACCACTTTGTACAAGAAAGCTGGGTTCggctgaatccgcatttccca	pFAST-R07 (Protein)
	ACO1-qRT-F	TTGAGTGAAGGCAAAACCTCAGATG	RT-qPCR (Lee *et al.*, 2017)
	ACO1-qRT-R	GCTGAGTTCCTCTGAAATGTTTGGG	
**ACO2**	pACO2-F	CAAGCTAAGCTTGAGCTCTATCAATTATTTCTCGTGGTTTTTTG	Gibson assembly to replace 35S in p2GWF7.0
	pACO2-R	TTTTTGTACAAACTTGTGATATCACTAGTCTTTCTTTCTCTCTCTCTTCTTTGA	
	ACO2- attB1-F	GGGGACAAGTTTGTACAAAAAAGCAGGCTTAatggagaagaacatgaagtttccag	BP: pZeo then modified p2GWF7.0
	ACO2- attB2r-R	GGGGACCACTTTGTACAAGAAAGCTGGGTTCgaaagtctctacggctgctgtag	
	ACO2-LP	GACAATCACAGCTGAGGAAGC	genotyping / sequencing
	ACO2-RP	TTAAACCGGAAGAACGACATG	
	ACO2-qRT-F	GCACCGTGTGGTGACTCAACA	RT-qPCR (Lee *et al.*, 2017)
	ACO2-qRT-R	AAGTCTCTACGGCTGCTGTAGGAT	
**ACO3**	pACO3-attB1-F	GGGGACAAGTTTGTACAAAAAAGCAGGCTTAgaggtctccgcattggggttg	BP: pDONR221 then
	pACO3-attB2-R	GGGGACCACTTTGTACAAGAAAGCTGGGTTCtctctctctctctcttaactagctact	pFASTG04 (Promoter)
	ACO3- attB2-R	GGGGGACCACTTTGTACAAGAAAGCTGGGTTCgaatgtctcaaccacagccacc	pFAST-R07 (Protein)
	ACO3-LP	ATCCCATCTCAAAAGCAGGAG	genotyping / sequencing
	ACO3-RP	CTTGAAACAGCAAAATGAGGC	
	ACO3-qRT-F	CAAGCATTCCATTGTCATCAACCTTG	RT-qPCR (Schellingen *et al.*, 2014)
	ACO3-qRT-R	TTTCTGGGTCATCACACGGTG	
	pACO3-1-attB4-F	GGGGACAACTTTGTATAGAAAAGTTGCTggaatttgtctcctcatcctgcta	BP: pDNOR221 P4-P1r,
	pACO3-1-attB1r-R	GGGGACTGCTTTTTTGTACAAACTTGTctctctctctctctcttaactagctact	

	pACO3-2-attB4-F	GGGGACAACTTTGTATAGAAAAGTTGCTttgtaattatattagctggccaagg	For Y1H-BAIT: pPMW#2, pMW#3
	pACO3-2-attB1r-R	GGGGACTGCTTTTTTGTACAAACTTGTtatttgaatcactatgaatagggaatgac	
	pACO3-3-attB4-F	GGGGACAACTTTGTATAGAAAAGTTGCTcgttaccattgaaagtaagtattttgttca	
	pACO3-3-attB1r-R	GGGGACTGCTTTTTTGTACAAACTTGTaactttgttaattttgggaaggaag	
	EcoRI-pACO3 fw	TCTATTATC GAATTC GAGGTCTCCGCATTGGGGTTG	LUC fusion
	NcoI-pACO3 rev	TCTATTATC CCATGG CTCTCTCTCTCTCTCTTAACTAGCTACT	
	A3_-2185_wt_s	TTGAGCCTTGAGATTATCGTATCTAGCTAGCTTCT	ACO3 oligos for DPI-ELISA
	A3_-2185_wt_a	AGAAGCTAGCTAGATACGATAATCTCAAGGCTCAA	
	A3_-2185_mut_s	TTGAGCCTTGACATTATGGTATGTAGCTAGCTTCT	
	A3_-2185_mut_a	AGAAGCTAGCTACATACCATAATGTCAAGGCTCAA	
	A3_-1585_wt_s	AATTAATTTCACAAGATACGAGAGAGATAAAGATTGATTTATTTTCTTGTTACTTGATACAAGATTTTTTGTGGCACGAA	
	A3_-1585_wt_a	TTCGTGCCACAAAAAATCTTGTATCAAGTAACAAGAAAATAAATCAATCTTTATCTCTCTCGTATCTTGTGAAATTAATT	
	A3_-1585_mut_s	AATTAATTTCACAACATACGAGAGACATAAACATTCATTTATTTTCTTGTTACTTCATACAACATTTTTTGTGGCACGAA	
	A3_-1585_mut_a	TTCGTGCCACAAAAAATGTTGTATGAAGTAACAAGAAAATAAATGAATGTTTATGTCTCTCGTATGTTGTGAAATTAATT	
**ACO4**	pACO4-attB1-F	GGGGACAAGTTTGTACAAAAAAGCAGGCTTAtccgcggattctatcttcgtacttgcat	BP: pDONR221 then
	pACO4-attB2-R	GGGGACCACTTTGTACAAGAAAGCTGGGTTCtctctctcttttttttaaatgggtttcttg	pFASTG04 (Promoter)
	ACO4- attB2-R	GGGGACCACTTTGTACAAGAAAGCTGGGTTCcgcagtggccaatggtcc	pFAST-R07 (Protein)
	pACO4-Seq1-F	GACTTCTCAAGTTGTTGTTTTGTA	sequencing
	ACO4-Seq2-R	AAAAGGTTACCTGTAATCGTCG	
	ACO4-LP	GTCCATATGCATTTGGACTGG	genotyping / sequencing
	ACO4-RP	GGAGCTACTGGATCTGCTGTG	
	ACO4-qRT-F	GAGTGCTATCTCAGACAGACGGAG	

ACO4-qRT-R	CTTGGTTCCTTGGCCTGAAACTTG	RT-qPCR (Lee *et al.*, 2017)
pACO4-1-attB4-F	GGGGACAACTTTGTATAGAAAAGTTGCTggaagaaaacgggtcaaacaatg	BP: pDNOR221 P4-P1r, For Y1H-BAIT: pPMW#2, pMW#3
pACO4-1-attB1r-R	GGGGACTGCTTTTTTGTACAAACTTGTtctctctctcttttttttaaatgggtttcttg	
pACO4-2-attB4-F	GGGGACAACTTTGTATAGAAAAGTTGCTataacaaagtatgaatgttgatcaaagaca	
pACO4-2-attB1r-R	GGGGACTGCTTTTTTGTACAAACTTGTgggtcggaaaaaatataaaaaatttatg	
pACO4-3-attB4-F	GGGGACAACTTTGTATAGAAAAGTTGCTaggaggtccactagtaggtcaagtt	
pACO4-3-attB1r-R	GGGGACTGCTTTTTTGTACAAACTTGTcattcattgccttacttcctttcta	
pACO4 attB1 AscI PacI SacI	GGGGACAAGTTTGTACAAAAAAGCAGGCTGGCGCGCCaaaTTAATTAAaaaaGAGCTCaggaggtccactagtaggtcaagtt	CFP transcription activation
pACO4 attB2 NLS	GGGGACCACTTTGTACAAGAAAGCTGGGTGcactttgcgcttcttcttgggCATCTCTCTCTCTTTTTTTTAAATGGGTTTCTTG	CFP transcription activation
venus YFP+NLS AscI Fw	AAAAGGCGCGCCatgcccaagaagaagcgcaaagtgGTGAGCAAGGGCGAG	CFP transcription activation
venus YFP PacI Rev	AAAATTAATTAActaCTTGTACAGCTCGTCCAT	CFP transcription activation
EcoRI-pACO4 fw	TCTATTATC GAATTC TCCGCGGATTCTATCTTCGTACTTGCAT	LUC fusion
NcoI-pACO4 rev	TCTATTATC CCATGG CTCTCTCTCTTTTTTTTTAAATGGGTTTCTTG	
A4_+89_wt_s	TTGAAGTTTGATATAAATCTGATGATCAAAGACTTG	ACO4 oligos for DPI ELISA
A4_+89_wt_a	CAAGTCTTTGATCATCAGATTTATATCAAACTTCAA	
A4_+89_mut_s	TTGAAGTTTGATATAAATCTGATGATCAAAGACTTG	
A4_+89_mut_a	CAAGTCTTTGATCATCAGATTTATATCAAACTTCAA	
A4_-1739_wt_s	TTGGTTTGGTAGATCTTTCTCCCTCGTGGCCTTGGGGTCAAGCTACAGCTTTGGTCAATCTCATGTTATCGACACCACAAA	
A4_-1739_wt_a		

	A4_-1739_mut_s	TTTGTGGTGTCGATAACATGAGATTGACCAAAGCTGTAGCTTGACC CCAAGGCCACGAGGGAGAAAGATCTACCAAACCAA	
	A4_-1739_mut_a	TTGGTTTGGTACATGTTTCTCCCTCGTGGCCTTGGGGTCAAGCTACA GCTTTGGTCAATGTCATGTTATGGACACCACAAA	
		TTTGTGGTGTCCATAACATGACATTGACCAAAGCTGTAGCTTGACCC CAAGGCCACGAGGGAGAAACATGTACCAAACCAA	
**ACO5**	pACO5-attB1-F	GGGGACAAGTTTGTACAAAAAAGCAGGCTTA cttgactagttggtatttacgctga	BP: pDONR221 then
	pACO5-attB2-R	GGGGACCACTTTGTACAAGAAAGCTGGGTTCttcagatccgcaaagagag aga	pFASTG04 (Promoter)
	ACO5-attB2-R	GGGGACCACTTTGTACAAGAAAGCTGGGTTCgagagactttacagctaga aaacga	pFAST-R07 (Protein)
	ACO5-GK-RP	CCTTTAGGCAAACCCAAATTC	genotyping
	ACO5-GK-LP	TGTAAGGGATTTCTGTTTCATCC	
	ACO5-qRT-F	TGTTCAGCCTCTACCTAATGCCA	RT-qPCR (Schellingen *et al.*, 2014)
	ACO5-qRT-R	CCTGTGCCACGCACTCTTGTA	
**ARR1**	arr1-3-F	CTTCAAGCACTAGCCGTCACAGGTCAGTT	genotyping
	arr1-3-R	AATGTTATCGATGGAGTATGCGTCAAAGT	
	ARR1-142aa-attB1-F	GGGGACAAGTTTGTACAAAAAAGCAGGCTTAatggaggcacttaagaac atatggc	BP: pDONR221 for Prey-pDEST22 (Y1H), pET-DEST42-GFP (DPI-ELISA)
	ARR1-Stop-attB2-R	GGGGACCACTTTGTACAAGAAAGCTGGGTTTCaaaccggaatgttatcga tgg	
	ARR1 Prom F-Mlu1	cccacgcgtgagtacagctgtgaaattgatggattactacc	Cloning of *pARR1::nls-2xGFP*
	ARR1 Prom R-EcoR1/EcoRV	cccGAATTCGATATCacctctctctatgtagctcgaacccaag	
	ARR1 3'UTR R-EcoR1/Stu1	cccgaattcaggcctttggataaaaaaaccgataaaccggagggact	
	ARR1 3'UTR R-Nhe1	cccgctagcTGGCAAGTCGTCTGCAACATCACTCAACCAAC	
	ARR1 GARP-attB1-F	GGGGACAAGTTTGTACAAAAAAGCAGGCTTAgcgtcgaatttgaagaaac cgcgtg	Cloning of ARR1 DNA binding domain. BP: pDONR221

	ARR1 GARP-STOP-attB2-R	GGGGACCACTTTGTACAAGAAAGCTGGGTTtcatccaagccgtcttagatatatcc	LR: pET-DEST42-GFP (DPI-ELISA)
**ARR2**	ARR2-5F	CCTTCTCTGATCGTTCGTTTTCTG	genotyping
	ARR2-5R	ATCAACGACAAGAACTCGAAGATTC	
	ARR2-125aa-attB1-F	GGGGACAAGTTTGTACAAAAAAGCAGGCTTAatggattacctcatcaaaccggtac	BP: pDONR221 for Prey-Y1H: pDEST22 & FLIM-FRET, pET-DEST42-GFP (DPI-ELISA)
	ARR2-stop-attB2-R	GGGGACCACTTTGTACAAGAAAGCTGGGTTTCagacctggatattatcgatggagta	
	ARR2 D80A fw	ggttttgatattgtcattagtgCtgttcatatgcctgacatgg	site-directed mutagenesis at D80A site.
	ARR2 D80A rev	ccatgtcaggcatatgaacaGcactaatgacaatatcaaaacc	
	ARR2-attB1-F	GGGGACAAGTTTGTACAAAAAAGCAGGCTTAatggtaaatccgggtcacggaag	Y2H and FLIM-FRET
	ARR2-attB2-R	GGGGACCACTTTGTACAAGAAAGCTGGGTTTCctagtctgagtttctgtagtcagacctgg	
**ARR10**	arr10-1-F	GCCACCTTCAGGTGAGAGTTAGACTATGAT	genotyping
	arr10-1-R	AGCTGACAAAGAAAAGGGAAAATGGAGTTT	
	ARR10-122aa-attB1-F	GGGGACAAGTTTGTACAAAAAAGCAGGCTTAatggaggagcttaagaacatatggc	BP: pDONR221 for Prey-pDEST22 (Y1H)
	ARR10-stop-attB2-R	GGGGACCACTTTGTACAAGAAAGCTGGGTTTCaagctgacaaagaaaagggaaaa	
**ARR12**	arr12-1-LP	CGGTACAATATGCGGATTTTGATTCGGTAT	genotyping
	arr12-1-RP	TAATAGCTTGCTGATTAGCCACACCACTGA	
	ARR12-122aa-attB1-F	GGGGACAAGTTTGTACAAAAAAGCAGGCTTAatggaggagttgaagaacatatggca	BP: pDONR221 for Prey-pDEST22 (Y1H)
	ARR12-stop-attB2-R	GGGGACCACTTTGTACAAGAAAGCTGGGTTTCatatgcatgttctgagtgaactaaac	
**EIN2-C**	EIN2-C-459aa-attB1-F	GGGGACAAGTTTGTACAAAAAAGCAGGCTTAatgacgccgctgaaatctgcga	BP: pDONR221 for prey-

	EIN2-C-attB2-R	GGGGGACCACTTTGTACAAGAAAGCTGGGTTTCaacccaatgatccgtacgcag	Y1H:pGAT7 and FLIM-FRET
**ACS2**	pACS2-attB1-F	GGGGACAAGTTTGTACAAAAAAGCAGGCTTAgacatgatcactgtgaagtcgtgc	BP pDONR221 for pFAST-R07
	ACS2-attB2-R	GGGGACCACTTTGTACAAGAAAGCTGGGTTCtgctcggagaagaggtgagtg	
	ACS2-LP	TTGTCGTTCAATTAAAACCGC	genotyping / sequencing
	ACS2-RP	AGAATTGACACAGCAAATGGG	
**ACS5**	ACS5-LP	CCAGCTATGTTTCGATCTAATCGAGTCATGGTTAAC	genotyping
	ACS5-RP	GAGGTCAAGCTCTGCTTCAAATGTGTTTGTGTCCA	
**ACS6**	ACS6-LP	CACTTGGTGAACAATCACACG	genotyping
	ACS6-RP	GCTTGCCTGAATTCAGACAAG	
**ACS7**	pACS7-attB1-F	GGGGACAAGTTTGTACAAAAAAGCAGGCTTAacgaattgtaaccaacacgcaatgg	BP pDONR221 for pFAST-R07
	ACS7-attB2-R	GGGGACCACTTTGTACAAGAAAGCTGGGTTCaaacctccttcgtcggtccat	
	ACS7-LP	AACTTGCTTTGTCCAAGCAAG	genotyping / sequencing
	ACS7-RP	ATCCTAACGACGCCCTTCTAG	
**ACS8**	ACS8-LP	ATAACCAACCCATCTAACCCG	genotyping
	ACS8-RP	GGCTTCTCAACCAGAAAGGTC	
**ACS9**	ACS9-LP	GTTTGAGAAGACACGAGACCG	genotyping
	ACS9-RP	CCTACTTCTTGGGATGGGAAG	
**UBC10**	UBC10-F	CAAGGTGCTGCTATCG	qRT-PCR
	UBC10-R	ATCTCGGGCACCAAAGG	
	HIS293RV	GGGACCACCCTTTAAAGAGA	pMW#2 &pMW#3 specific primers.
	LacZ592RV	ATGCGCTCAGGTCAAATTCAGA	
**NLS:RFP**	**nls:RFP-attB1-F**	GGGGACAAGTTTGTACAAAAAAGCAGGCTTACCCAAGAAGAAGCGCAAAGTGATGGCCTCCTCCGAGGAC	Positive control for FLIM-FRET assay.
	**RFP-attB2-R**	GGGGACCACTTTGTACAAGAAAGCTGGGTTTAGGCGCCGGTGGAGTG	

Supplemental References

Bates, D., Machler, M., Bolker, B.M., and Walker, S.C. (2015). Fitting Linear Mixed-Effects Models Using lme4. J Stat Softw **67**:1-48. DOI 10.18637/jss.v067.i01.

Brady, S.M., Orlando, D.A., Lee, J.Y., Wang, J.Y., Koch, J., Dinneny, J.R., Mace, D., Ohler, U., and Benfey, P.N. (2007). A high-resolution root spatiotemporal map reveals dominant expression patterns. Science **318**:801-806. 318/5851/801 [pii]

10.1126/science.1146265.

Brand, L.H., Kirchler, T., Hummel, S., Chaban, C., and Wanke, D. (2010). DPI-ELISA: a fast and versatile method to specify the binding of plant transcription factors to DNA in vitro. Plant Methods **6**:25. 10.1186/1746-4811-6-25.

Castrillo, G., Turck, F., Leveugle, M., Lecharny, A., Carbonero, P., Coupland, G., Paz-Ares, J., and Onate-Sanchez, L. (2011). Speeding cis-trans regulation discovery by phylogenomic analyses coupled with screenings of an arrayed library of Arabidopsis transcription factors. PLoS One **6**:e21524. 10.1371/journal.pone.0021524.

French, A.P., Wilson, M.H., Kenobi, K., Dietrich, D., Voss, U., Ubeda-Tomas, S., Pridmore, T.P., and Wells, D.M. (2012). Identifying biological landmarks using a novel cell measuring image analysis tool: Cell-o-Tape. Plant Methods **8**:7. 10.1186/1746-4811-8-7.

Horak, J., Grefen, C., Berendzen, K.W., Hahn, A., Stierhof, Y.D., Stadelhofer, B., Stahl, M., Koncz, C., and Harter, K. (2008). The Arabidopsis thaliana response regulator ARR22 is a putative AHP phospho-histidine phosphatase expressed in the chalaza of developing seeds. BMC Plant Biol **8**:77. 1471-2229-8-77 [pii]

10.1186/1471-2229-8-77.

Lee, H.Y., Chen, Y.C., Kieber, J.J., and Yoon, G.M. (2017). Regulation of the turnover of ACC synthases by phytohormones and heterodimerization in Arabidopsis. Plant J **91**:491-504. 10.1111/tpj.13585.

Pacheco-Escobedo, M.A., Ivanov, V.B., Ransom-Rodriguez, I., Arriaga-Mejia, G., Avila, H., Baklanov, I.A., Pimentel, A., Corkidi, G., Doerner, P., Dubrovsky, J.G., et al. (2016). Longitudinal zonation pattern in Arabidopsis root tip defined by a multiple structural change algorithm. Ann Bot **118**:763-776. 10.1093/aob/mcw101.

R_Core_Team (2021). R: A language and environment for statistical computing. R Foundation for Statistical Computing, Vienna, Austria. URL: https://www.R-project.org/.

Reece-Hoyes, J.S., and Walhout, A.J. (2012). Gene-centered yeast one-hybrid assays. Methods Mol Biol **812**:189-208. 10.1007/978-1-61779-455-1_11.

Rieger, J., Fitz, M., Fischer, S.M., Wallmeroth, N., Flores-Romero, H., Fischer, N.M., Brand, L.H., Garcia-Saez, A.J., Berendzen, K.W., and Mira-Rodado, V. (2023). Exploring the Binding Affinity of the ARR2 GARP DNA Binding Domain via Comparative Methods. Genes (Basel) **14**10.3390/genes14081638.

Sakai, H., Aoyama, T., and Oka, A. (2000). Arabidopsis ARR1 and ARR2 response regulators operate as transcriptional activators. The Plant journal : for cell and molecular biology **24**:703-711.

Salazar, C., Armenta, J.M., Cortes, D.F., and Shulaev, V. (2012). Combination of an AccQ.Tag-ultra performance liquid chromatographic method with tandem mass spectrometry for the analysis of amino acids. Methods Mol Biol **828**:13-28. 10.1007/978-1-61779-445-2_2.

Schellingen, K., Van Der Straeten, D., Vandenbussche, F., Prinsen, E., Remans, T., Vangronsveld, J., and Cuypers, A. (2014). Cadmium-induced ethylene production and responses in Arabidopsis thaliana rely on ACS2 and ACS6 gene expression. BMC Plant Biol **14**:214. 10.1186/s12870-014-0214-6.

Voinnet, O., Lederer, C., and Baulcombe, D.C. (2000). A Viral Movement Protein Prevents Spread of the Gene Silencing Signal in Nicotiana benthamiana. Cell **103**:157-167. 10.1016/s0092-8674(00)00095-7.

Zdarska, M., Cuyacot, A.R., Tarr, P.T., Yamoune, A., Szmitkowska, A., Hrdinova, V., Gelova, Z., Meyerowitz, E.M., and Hejatko, J. (2019). ETR1 Integrates Response to Ethylene and Cytokinins into a Single Multistep Phosphorelay Pathway to Control Root Growth. Mol Plant **12**:1338-1352. 10.1016/j.molp.2019.05.012.
